# Supplementary material for: Violet/ultraviolet light-induced depassivation in halide perovskite solar cells
Source: Nat Commun. 2025 Dec 17;16:11409. doi: 10.1038/s41467-025-66227-4 (PMC12739151; doi:10.1038/s41467-025-66227-4)
Supplement: Supplementary file 1 — Supplementary Information [file 41467_2025_66227_MOESM1_ESM.pdf]

# Supplementary Information

## Violet/ultraviolet light-induced depassivation in halide perovskite solar cells

Dejian Yu[1]†, Fei Cao[1]†, Xiaosong Qiu[1]†, Ying Chen[1]†, Zhipeng Zhang[1], Gang Wang[1], Yulin Mao[1], Junwen Zhong[2]*, Chenliang Su[3]*, Wei Huang[4], Tae-Woo Lee[5]*, Guichuan Xing[1]*

[1]Institute of Applied Physics and Materials Engineering, University of Macau; Macau, 999078, China.

[2]Department of Electromechanical Engineering, Faculty of Science and Technology, University of Macau; Macau, 999078, China.

[3]International Collaborative Laboratory of 2D Materials for Optoelectronics Science and Technology of Ministry of Education, Institute of Microscale Optoelectronics, Shenzhen University; Shenzhen, 518060, China.

[4]MIIT Key Laboratory of Flexible Electronics (KLoFE), Institute of Flexible Electronics (IFE), Northwestern Polytechnical University (NPU); Xi'an, 710129, China

[5]Department of Materials Science and Engineering, Interdisciplinary Program in Bioengineering, Institute of Engineering Research, Research Institute of Advanced Materials, Soft Foundry, Seoul National University, Seoul 08826, Republic of Korea.

*Corresponding author. Email: gcxing@um.edu.mo; twlees@snu.ac.kr; chmsuc@szu.edu.cn; junwenzhong@um.edu.mo

†These authors contributed equally to this work.

## Supplementary Notes

**Supplementary Note #1:** The density functional theory (DFT) simulation was conducted to investigate the adsorption behavior of $H^+$ and $I^-$ species on the $BA_2PbI_4$ surface. Various initial adsorption positions (shown in fig. S23a) were explored to identify the most energetically favorable sites for the $H^+$ cation and $I^-$ anion.

As depicted in Fig. S23b, the $I^-$ anion is preferentially adsorbed near the ammonium group (site 2, the nitrogen atom of the ammonium group), forming an R-$NH_3$...I (R denotes alkyl or aryl groups) hydrogen bond. The calculated bond length of 2.28 Å falls within the typical range of 2.20–2.50 Å for strong hydrogen bonds,[1] indicating a robust interaction between the $I^-$ anion and the ammonium group.

The nitrogen atom of the ammonium group is also the preferred site for the $H^+$ cation. Additionally, although multiple initial adsorption positions were considered in the simulations, the final relaxed structures consistently showed that the $H^+$ cation would be fully transferred away from the R-$NH_3^+$ group, resulting in the formation of $H_2$ and a deprotonated butylammonium (BA) organic species (fig. S23c).

**Supplementary Note #2:** The stability of the 2D halide perovskites (HPs) in the hydroiodic acid (termed HI hereafter) solution throughout the test period is crucial for ensuring the reliability of the photoreaction experiment. Previous studies reported that the HI solution typically requires saturation to prevent further dissolution of the HP materials.[2-4]

The DFT results reveal that, regardless of the alkyl chain length of the organic ligand, the nitrogen atom of the ammonium group serves as the active site. This finding allows us to adopt water-stable 2D HPs for the experiment. In this work, we employ the $ODA_2PbI_4$ compound, where $ODA^+$ represents the octadecylammonium cation. Notably, in contrast to previous reports, $ODA_2PbI_4$ in this study does not require a saturated solution for protection, as it is inherently stable against the highly acidic and polar HI solution. Furthermore, the $ODA_2PbI_4$ sample is prepared in the form of nanoflakes, which exhibit excellent dispersibility in the HI solution.

To verify the robustness of $ODA_2PbI_4$ for the photoreaction experiment, we systematically assessed the emissive properties of $ODA_2PbI_4$ nanoflakes dispersed in the HI solution and the benign toluene solvent, respectively. Fig. S24a presents the PL spectra of the two dispersions. The photoluminescence (PL) profile of the HI dispersion does not exhibit any alteration, suggesting negligible degradation of the $ODA_2PbI_4$ caused by the HI solution, as this would otherwise lead to broadening, emergence of side peaks, or exacerbated asymmetry in the PL spectrum. Furthermore, we explored the excitation dependence of the PL for both samples. As shown in the inset of fig. S24b, the $ODA_2PbI_4$ dispersed in the HI solution demonstrates the same linear relationship between PL intensity and excitation power as the sample in the air, corroborating that the $ODA_2PbI_4$ can well preserve its excitonic recombination dynamics when in the HI solution. Subsequently, we measured the carrier lifetimes of the samples in the two solvents using time-

resolved PL (TRPL) spectroscopy, as presented in fig. S24b. Interestingly, we found that the PL lifetime of the $ODA_2PbI_4$ in the HI solution is even slightly longer than that of the sample dispersed in the benign toluene solvent (0.73 ns vs. 0.70 ns). This observation suggests that the harsh HI environment does not introduce mid-gap states but rather suppresses non-radiative recombination pathways, which might be attributed to the intensified protonation of the $ODA^+$ cation in the HI solution, leading to a more ordered $ODA^+$-$I^-$ bonding arrangement.

The robustness of the $ODA_2PbI_4$ HP material was further confirmed with structural analysis. Even after 6 hours of photoreaction in the HI solution, scanning electron microscope (SEM) imaging revealed no discernible changes in the morphology of the $ODA_2PbI_4$ nanoflakes (fig. S24c, under Xenon lamp illumination of AM 1.5 G in the HI solution). Moreover, the X-ray diffraction (XRD) pattern of the $ODA_2PbI_4$ sample remained phase-pure, as shown in fig. S24d. These structural characterizations corroborate the exceptional stability of the $ODA_2PbI_4$ material under the harsh conditions of the HI solution and prolonged photoreaction.

The ultraviolet photoelectron spectroscopy (UPS) characterization was also conducted to analyze the electrical structure cleanliness of $ODA_2PbI_4$ before and after the hydrogen evolution reaction. The sharpness of the Fermi level edge and the secondary electron edge (SEE) in the UPS spectrum reflects the degree of electronic order near the valence band maximum (VBM) and the Fermi level, respectively (fig. S24e). As shown in fig. S24f and fig. S24g, after the photoreaction, the SEE level edge and the Fermi level edge remained as sharp as before the photoreaction. This suggests that the long-term operation of the device introduces negligible trap states or defects into the electronic structure of the $ODA_2PbI_4$.

The above results show that $ODA_2PbI_4$ inherently possesses high structural and functional robustness for stable operation in the HI solution.

**Supplementary Note #3:** We found the energy landscape of the 2D HP/HI interface allows for the photocatalytic generation of $H_2$ driven by the direct carrier transfer from the HP lattice to the $H^+/I^-$ ions in the solution, which explains the residual $H_2$ generation upon visible illumination.

According to the Mott-Schottky analysis shown in fig. S25a, the Fermi-level position of $ODA_2PbI_4$ is located at -0.62 eV relative to the normal hydrogen electrode (NHE). This Fermi-level position corresponds to a work function of 3.88 eV. The X-ray photoelectron spectroscopy (XPS) valence band spectrum in fig. S25b reveals an ionization potential (the energy gap between the VBM and the vacuum level) of 5.48 eV for $ODA_2PbI_4$. Based on the established bandgap of 2.48 eV for $ODA_2PbI_4$, the conduction band minimum (CBM) can be inferred to be -3.00 eV relative to the vacuum level.

To investigate the redox potentials of the HI solution, we conducted capacitance-voltage (C-V) measurements. The resulting C-V curve presented in fig. S25c reveals that the redox potentials of splitting HI into $H_2$ and $I_3^-$ are 0.49 eV and 0.71 eV versus the NHE, respectively.

Combining these experimentally determined redox potentials with the electronic structure parameters of $ODA_2PbI_4$, we can construct a detailed flat-band energy diagram depicting the energetic landscape at the interface between the $ODA_2PbI_4$ and the HI solution, as shown in fig. S25d. Indeed, this energy alignment allows the photocatalytic generation of $H_2$ arising from the direct carrier transfer from the HP lattice to the $H^+/I^-$ ions in the solution.

**Supplementary Note #4:** In solid-state HP solar cells, there would be no re-protonation process as in the solution-phase photoreaction system, as there is no free $H^+$. Consequently, deprotonated ammonium species are anticipated to debond from the HP framework, resulting in the degradation of 2D HPs.

To verify this hypothesis, we irradiated 2D HP $BA_2PbI_4$ films with monochromatic light at ~2.76 eV (450 nm) nm and ~3.40 eV (365 nm), respectively. The ~2.76 eV irradiation selectively excites the excitonic transition, while the ~3.40 eV irradiation can excite the ammonium-to-HP carrier transition (fig. S26a). The irradiation intensity from both sources was carefully controlled to 20 mW $cm^{-2}$. The absorbed photon fluxes in the HP films were $4.22\times10^{16}$ photons $s^{-1}$ $cm^{-2}$ for the ~2.76 eV irradiation and $3.40\times10^{16}$ photons $s^{-1}$ $cm^{-2}$ for the ~3.40 eV irradiation.

As shown in fig. S26b, the absorption profile of the $BA_2PbI_4$ film under ~2.76 eV irradiation remained stable within the first 60 min, indicating a high photostability when subjected to excitonic photoexcitation. In stark contrast, despite a lower photon absorption flux, the ~3.40 eV irradiation caused a conspicuous decline in the resonance intensity (fig. S26c) after 60 minutes of exposure. Concurrently, the absorption edge also became blunted after this irradiation treatment, implying the appearance of mid-gap trap states.[5]

Using the Urbach energy as a quantitative metric, the ~2.76 eV and ~3.40 eV irradiations were found to impose markedly different degradation kinetics on the 2D HP film. As compared in fig. S26d, the degradation upon the ~3.40 eV irradiation started immediately and featured a short characteristic time ($\tau$) of 12.2 minutes. In contrast, a discernible degradation upon the ~2.76 eV irradiation only appeared after 60 minutes and featured a much longer $\tau$ of 55.6 minutes. This contrast in the characteristic time indicates that the two irradiation conditions trigger distinct decomposition pathways in the 2D HP material. The mild photolysis induced by the excitonic

photoexcitation was previously unveiled to arise from a photooxidation process.[6] On the other hand, the acute photodegradation under the ~3.40 eV irradiation could be attributed to light-activated deprotonation and detachment of the ammonium species. Indeed, the XRD pattern reveals the emergence of $PbI_2$ following the UV irradiation (fig. S26e). Noteworthily, environmental factors caused negligible interference, as the Urbach energy of the $BA_2PbI_4$ film remained unvaried in the dark.

**Supplementary Note #5:** It should be noted that UV lights affect HPs in several ways. Previous reports showed that long-term, continuous UV irradiation would result in irreversible chemical decomposition of HPs.[7, 8] Short-term illumination would generate metastable mid-gap states in HPs, but these metastable states could disappear after a self-healing process in darkness.[9, 10] To highlight the mechanism proposed here, the testing method needs to minimize interference by other parallel impact mechanisms. Therefore, we adopted the short-term, alternating illumination/darkness test method proposed by Motti et al. with modifications.[11]

In detail, the solar cell was subjected to a short-term (7 s) illumination followed by a recovery process in darkness (3 s). During illumination, a defect annihilation process and a defect generation process would be triggered simultaneously:

1) The photo-driven annihilation of Frenkel defects ($I_i^-$-$V_I^+$ Frenkel pairs) that enhances the photovoltaic performance.[11, 12]

2) The reaction between two filled traps $I_i^0$ to form one $I_2$ molecule that compromises the photovoltaic performance.[11]

The defect generation process tends to occur at a defective region where the $I^-$ is poorly stabilized, but can be suppressed upon passivation by ammonium-terminated ligands. The two processes are ion-migration-related, thus are cumulative and barely reversible in darkness.[11] Therefore, the alternating testing can minimize the interference from parallel mechanisms.

UV lights could also jeopardize self-assembled monolayers (SAMs), decomposing their aromatic rings and weakening the interaction between them and ITO.[13] However, the harm to SAMs by UV irradiations only becomes discernible in hours or tens of hours,[13, 14] while in our test protocol with intermittent light-dark alternation, the solar cell in every cycle was first illuminated for an current-voltage (I-V) scan that lasts 7 seconds, and was then allowed to recover in the dark for 3 seconds for self-healing. A significant distinction in performance degradation was observed after 47 cycles (as shown in Figure 4E in the main text), amounting to only 329 s of irradiation. During this time slot, the impact of UV lights on the SAM layer could be minimal.

Based on the above discussion, the texting protocol here allows effective isolation of the light-induced depassivation effect on HP solar cells.

**Supplementary Note #6:** To learn the photodegradation pattern of the passivated solar cell, the fatigue test was conducted in a dry ambient airflow with the (unencapsulated) cells subjected to the two illumination conditions. During the tests, the solar cells operated in an open-circuit condition, and we collected performance data at regular intervals.

We observed that the degradation of the HP solar cells was significantly faster than previous reports on similar cell configurations and HP compositions. After 24 hours, the solar cell under unfiltered illumination exhibits a significant decline in open-circuit voltage ($V_{oc}$) to 0.906 V and fill factor (FF) to 57.15%, while the device under filtered illumination retains a much higher $V_{oc}$ of 1.103 V and an FF of 64.72%. The residual short-circuit current ($J_{sc}$) values are less distinct between the two sets of solar cells, measuring 19.23 mA/cm$^2$ for the unfiltered and 20.50 mA/cm$^2$ for the filtered illumination.

Assuming that $V_{oc}$ is primarily determined by the quality of the HP absorber layer, it would be greatly affected by the non-radiative recombination rate within the HP layer, as described by the following formula:[15]

$$V_{oc} \approx V_{oc}^{SQ} + \mathrm{k}T/\mathrm{q}\ \mathrm{In}(R_{rad} / (R_{rad} + R_{non\text{-}rad})) \qquad (1)$$

where $V_{oc}^{SQ}$ is the $V_{oc}$ in the Shockley-Queisser limit in which only interband radiative recombination occurs. q denotes the magnitude of the elementary charge. $R_{rad}$ and $R_{non\text{-}rad}$ denote radiative recombination rate and non-radiative recombination rate, respectively. k is Boltzmann constant, and T is temperature. Thus, the significant $V_{oc}$ decline observed in the device under the

unfiltered illumination suggests a dramatic increase in defect density, which also explains the decline in FF.

In contrast, $J_{sc}$ is usually less affected by defects. Accordingly, these results indicate a significantly higher defect density in the HP layer exposed to the unfiltered illumination, accounting for the photodegradation.

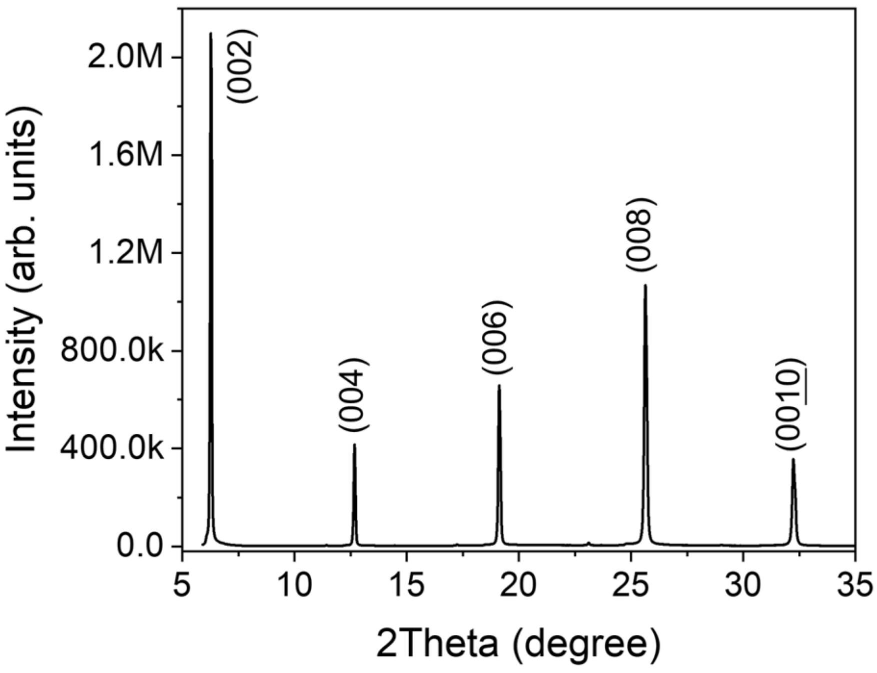


**Fig. S1. XRD pattern of the $BA_2PbI_4$ film.** The XRD pattern measured for the $BA_2PbI_4$ film shows characteristic diffraction peaks that well match those of a single-crystalline $BA_2PbI_4$ reported previously,[16] indicating high phase purity.

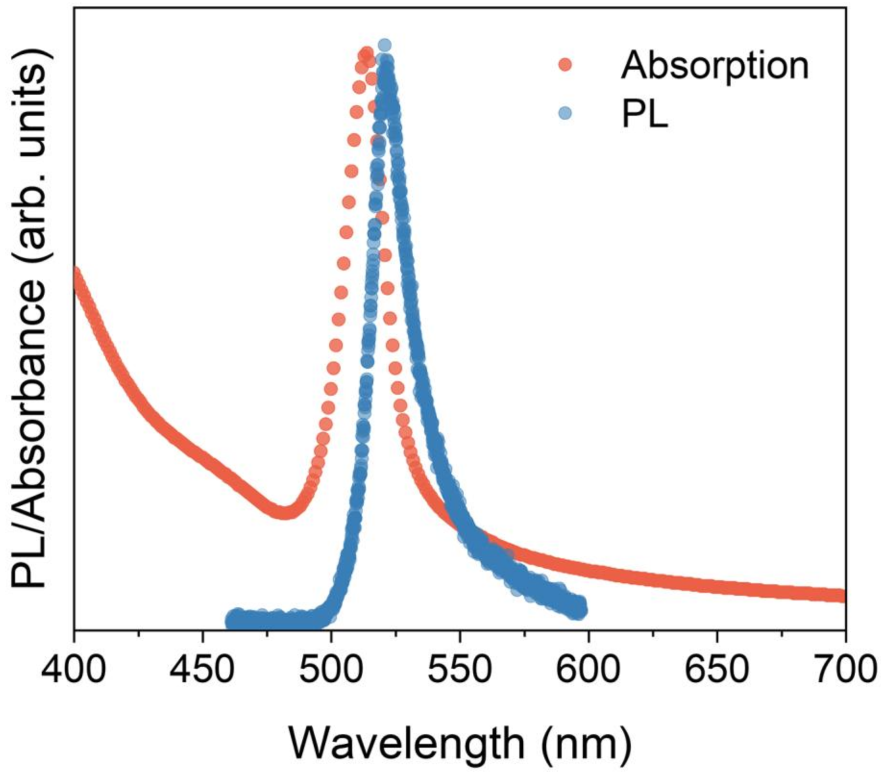


**Fig. S2. The spectral characterization of the $BA_2PbI_4$ film.** Steady-state PL and absorption spectra of the 2D HP $BA_2PbI_4$ film, which serves as the testbed for mechanism exploration in this work.

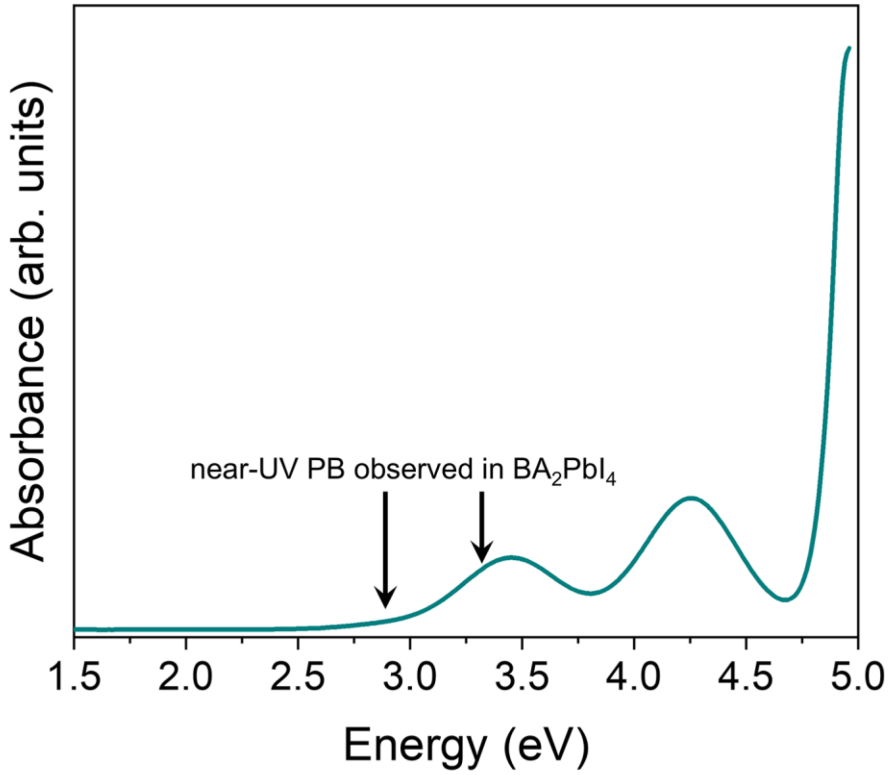


**Fig. S3. Absorption spectrum of BAI.** The absorption spectrum measured for BAI does not match the observed near-UV photobleaching (PB) signals in the transient absorption (TA) spectrum of $BA_2PbI_4$, thus ruling out the possibility that the near-UV PB signals arise due to the potential inclusion of BAI. The absorption onset of $PbI_2$ is 2.3 - 2.4 eV,[17] which does not match, either.

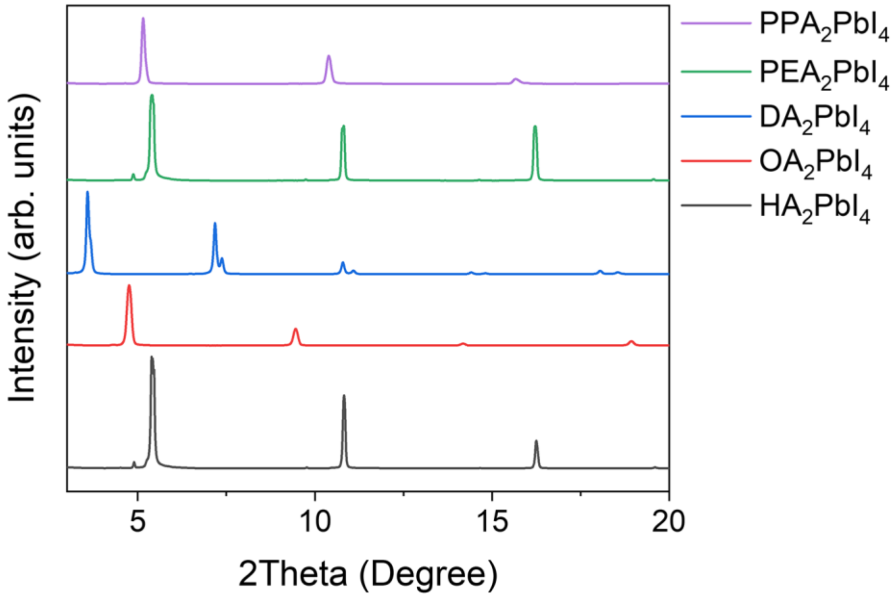


**Fig. S4. XRD patterns of varying 2D HP films adopted.** XRD patterns of a series of 2D HPs (including $PPA_2PbI_4$, $PEA_2PbI_4$, $DA_2PbI_4$, $OA_2PbI_4$, and $HA_2PbI_4$, where PPA, PEA, DA, OA, and HA denote phenylpropylammonium, phenylethylammonium, dodecylammonium, octylammonium, and hexylammonium, respectively) films. Noteworthily, the XRD pattern of $DA_2PbI_4$ shows a peak splitting, which we believe arises from micro-strain within the film that leads to less defined interlayer spacing. The micro-strain is due to a super high hydrophobicity of DA (because of its ultra-long alkyl side chain) that results in relatively poor film quality. We believe this is why the near-UV PB signal of the $DA_2PbI_4$ film in fig. S7 is less conspicuous (but discernible).

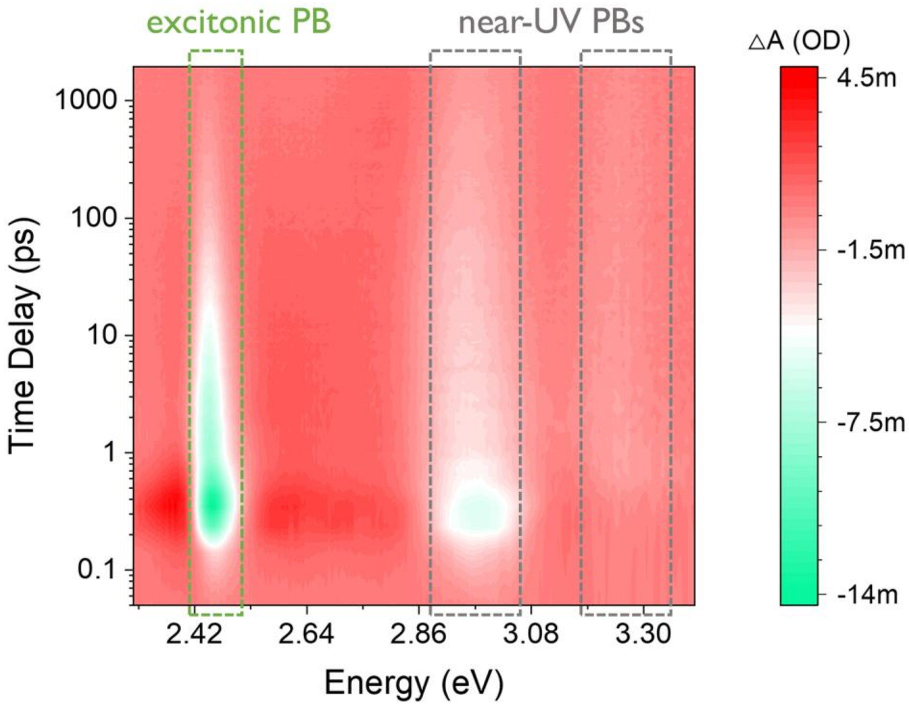


**Fig. S5. The TA spectrum of $PPA_2PbI_4$.** Pseudocolor ultrafast TA spectrum of the $PPA_2PbI_4$ film under a 3.82 eV (325 nm, 1 kHz, 100 fs, ~ 2.29 mW/cm$^2$) excitation light. A near-UV PB signal can be observed in addition to the excitonic PB signal.

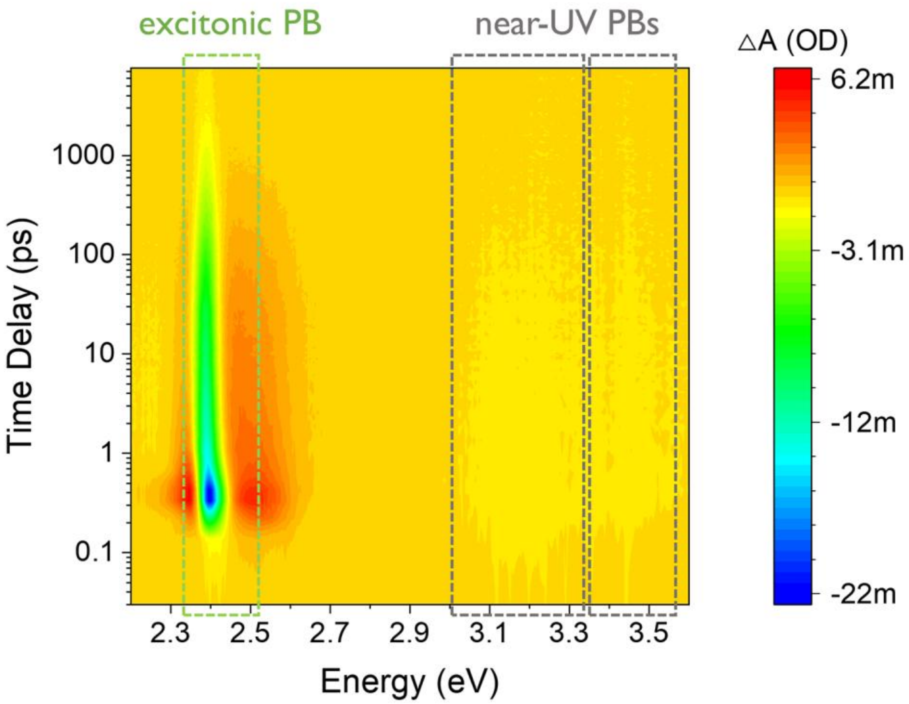


**Fig. S6. The TA spectrum of $PEA_2PbI_4$.** Pseudocolor ultrafast TA spectrum of the $PEA_2PbI_4$ film under a 3.82 eV (325 nm, 1 kHz, 100 fs, ~ 0.318 mW/cm$^2$) excitation light. A near-UV PB signal can be observed in addition to the excitonic PB signal.

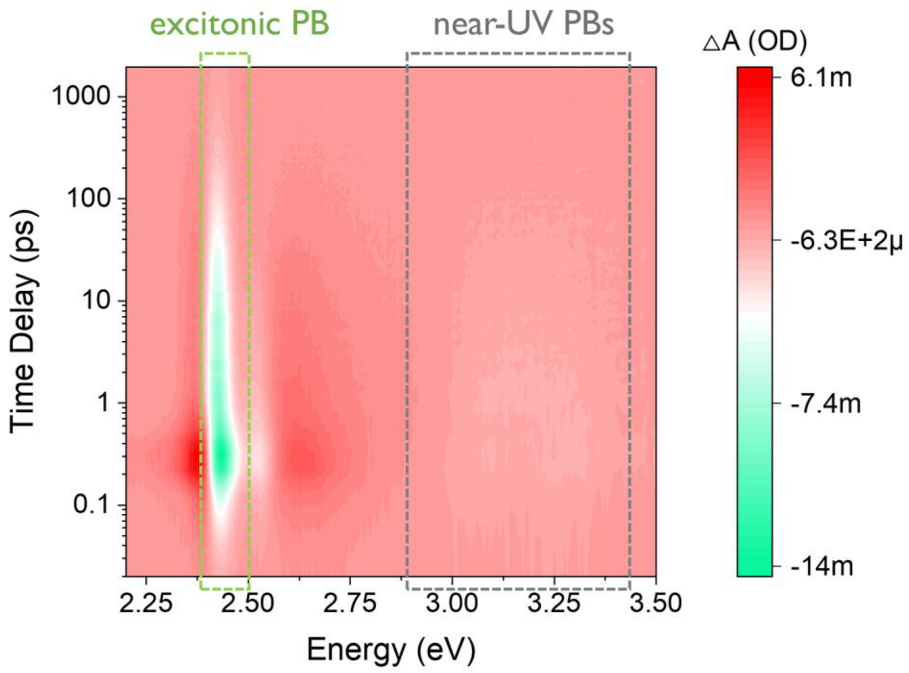


**Fig. S7. The TA spectrum of $DA_2PbI_4$.** Pseudocolor ultrafast TA spectrum of the $DA_2PbI_4$ film under a 3.82 eV (325 nm, 1 kHz, 100 fs, ~ 2.29 mW/cm$^2$) excitation light. A near-UV PB signal can be observed in addition to the excitonic PB signal. The near-UV PB signal of the $DA_2PbI_4$ film is relatively weak (but discernible) due to inadequate film quality, as introduced in the figure caption of fig. S4.

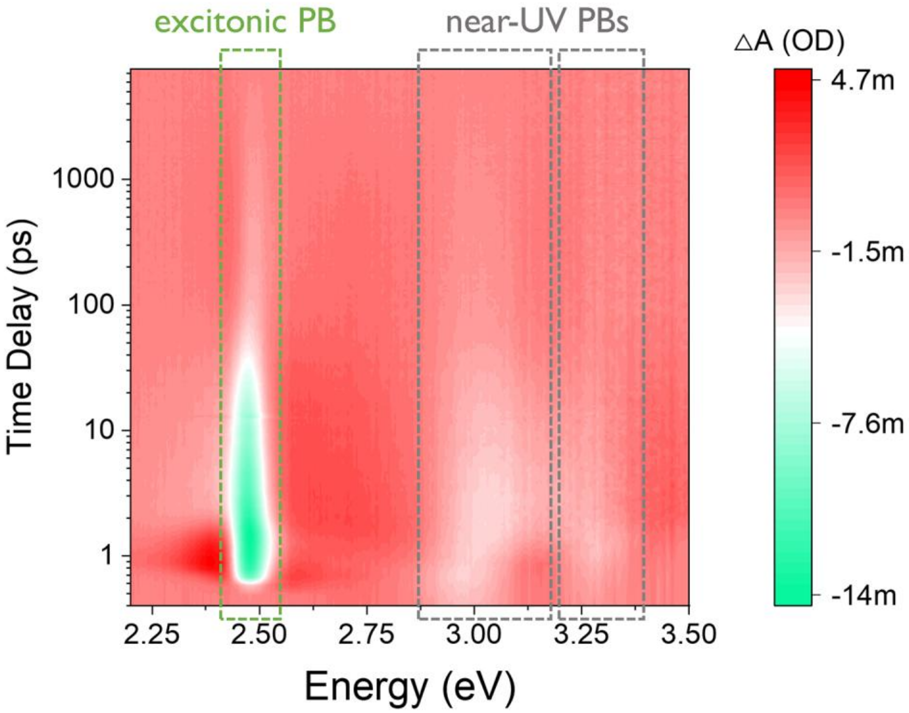


**Fig. S8. The TA spectrum of $OA_2PbI_4$.** Pseudocolor ultrafast TA spectrum of the $OA_2PbI_4$ film under a 3.82 eV (325 nm, 1 kHz, 100 fs, ~ 2.095 $mW/cm^2$) excitation light. A near-UV PB signal can be observed in addition to the excitonic PB signal.

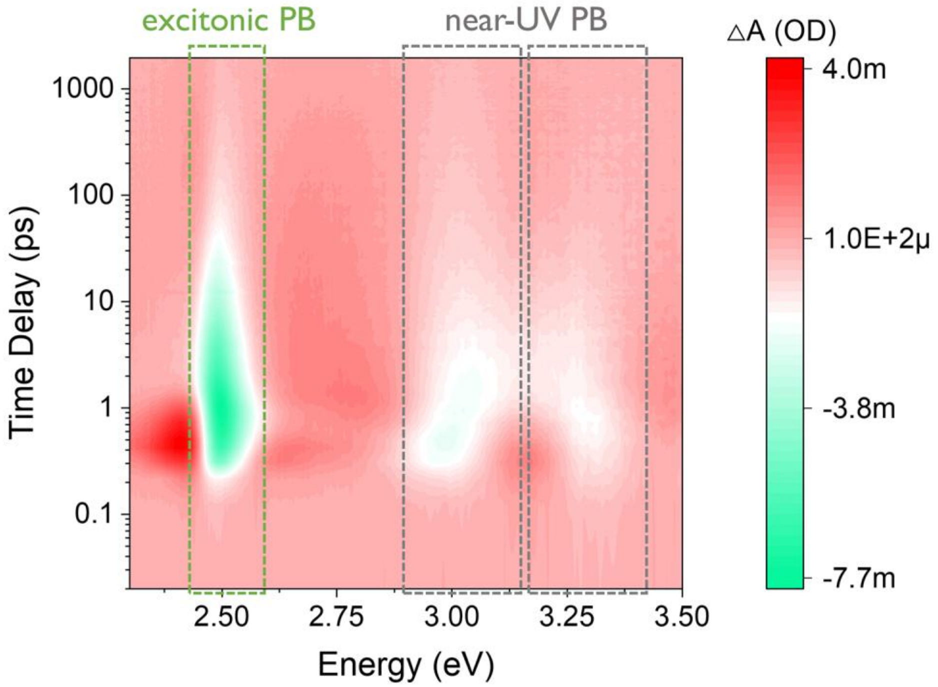


**Fig. S9. The TA spectrum of $HA_2PbI_4$.** Pseudocolor ultrafast TA spectrum of the $HA_2PbI_4$ film under a 3.82 eV (325 nm, 1 kHz, 100 fs, ~ 1 mW/cm$^2$) excitation light. A near-UV PB signal can be observed in addition to the excitonic PB signal.

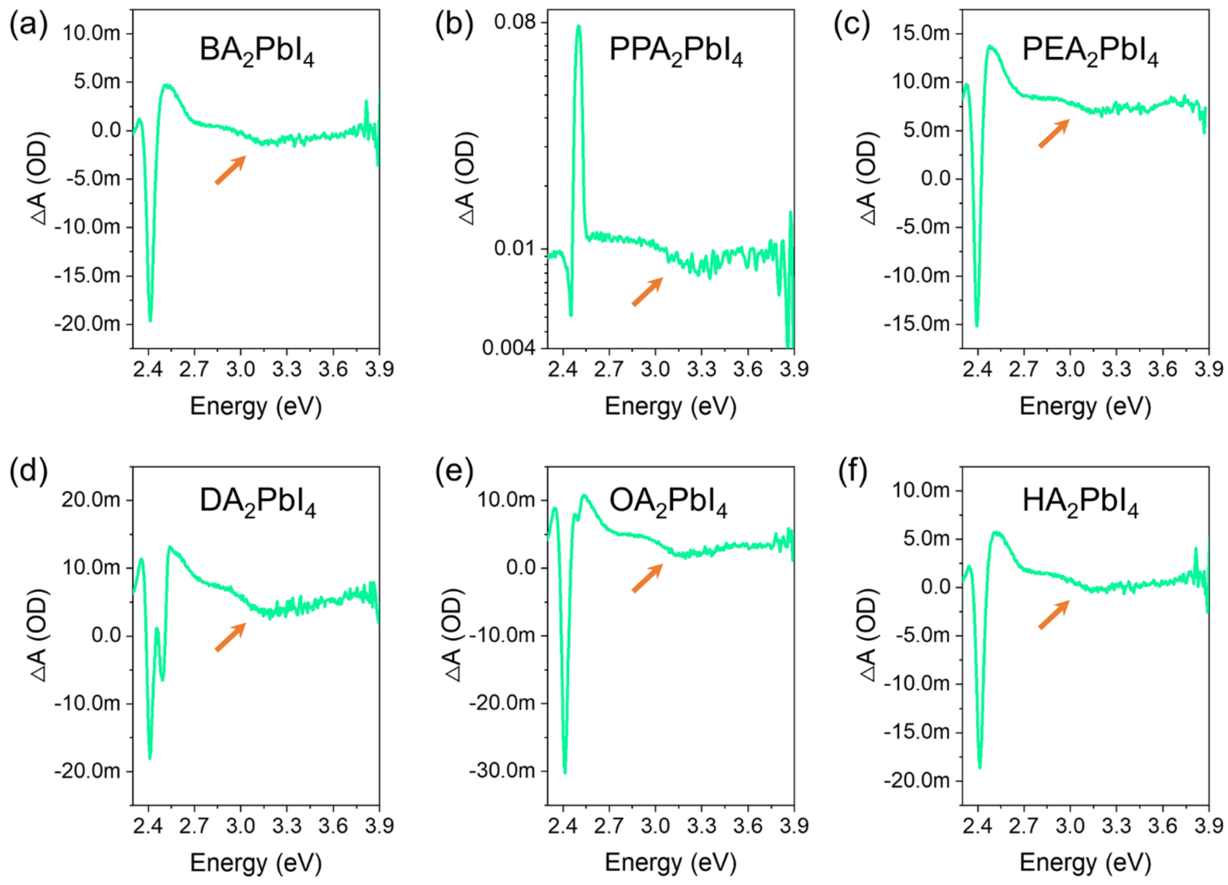


**Fig. S10. TA spectra of different 2D HPs upon quasi-resonance excitation.** TA spectra of different 2D HPs upon quasi-resonance excitation by the 2.48 eV excitation light. The 2D HPs in sequential are (a) $BA_2PbI_4$, (b) $PPA_2PbI_4$, (c) $PEA_2PbI_4$, (d) $DA_2PbI_4$, (e) $OA_2PbI_4$, and (f) $HA_2PbI_4$, respectively.

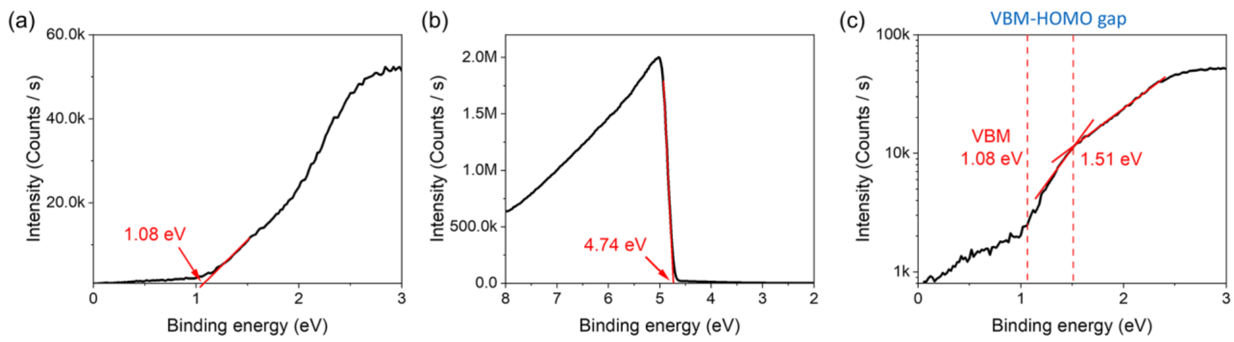


**Fig. S11. UPS characterization of the $BA_2PbI_4$ film.** (a) Fermi region and (b) secondary electron region of the UPS spectrum of $BA_2PbI_4$. (c) Identification of the possible VBM-HOMO (HOMO denotes the highest occupied molecular orbital) energy gap in the logarithmic scale of the Fermi region of the UPS spectrum.

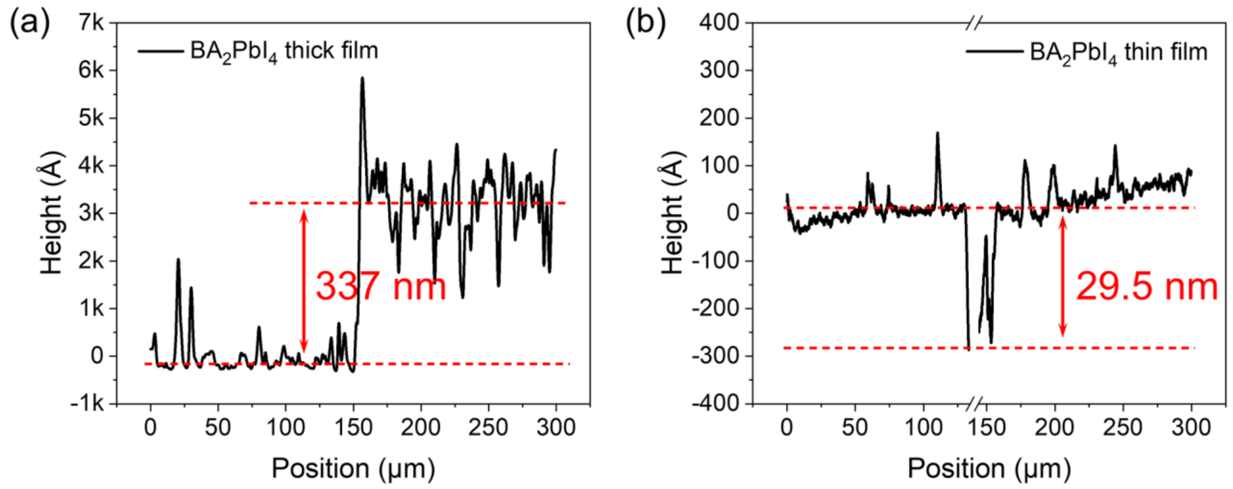


**Fig. S12. Thickness characterizations of thick and thin $BA_2PbI_4$ films.** Thickness measurements of (a) the thick $BA_2PbI_4$ film and (b) the thin $BA_2PbI_4$ film, obtained using a step profiler.

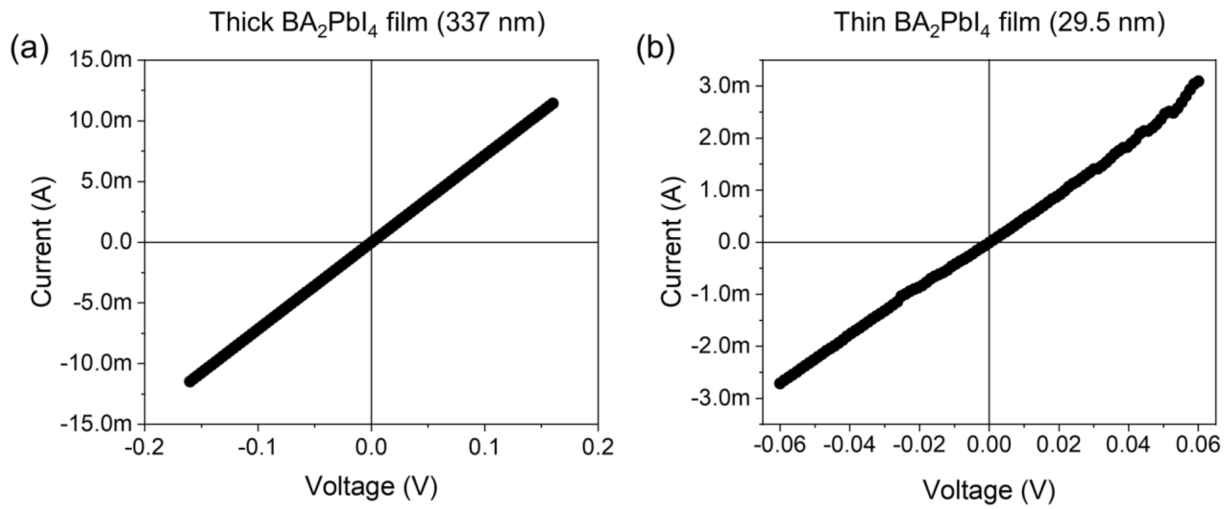


**Fig. S13. I-V characteristics of devices with the configuration ITO/$BA_2PbI_4$/Cu.** The thicknesses of the $BA_2PbI_4$ films for the two devices are (a) 337 nm and (b) 29.5 nm, respectively. Both devices exhibit ohmic contact properties.

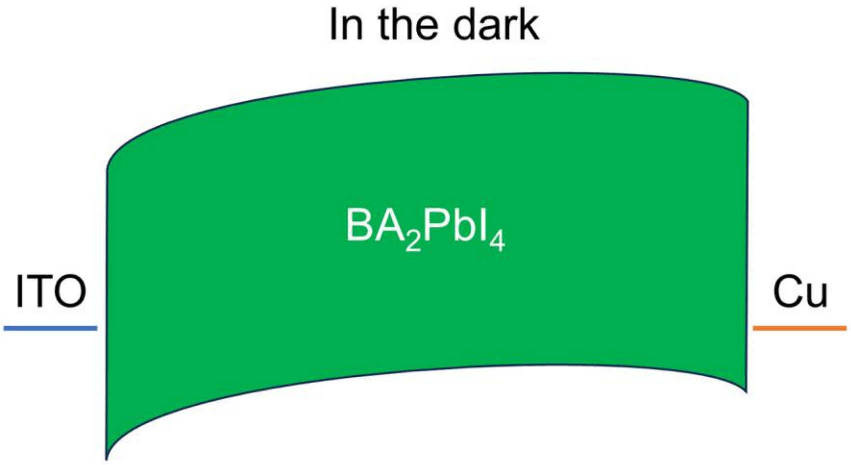


**Fig. S14. Energy landscape across the ITO/$BA_2PbI_4$/Cu device in the dark.** Schematic illustration of the built-in potentials in the HP layer at the near-Cu side and the near-ITO side. Due to the work function distinction between ITO and Cu electrodes, the built-in potential at the two sides would be different, which accounts for the exponential ($I_{for}$+$I_{rev}$) versus $V_{abs}$ characteristic of the ITO/$BA_2PbI_4$ (thick film)/Cu device in the dark. $I_{for}$, $I_{rev}$, and $I_{abs}$ denote the current in the forward direction, the current in the reverse direction, and the absolute bias regardless of direction.

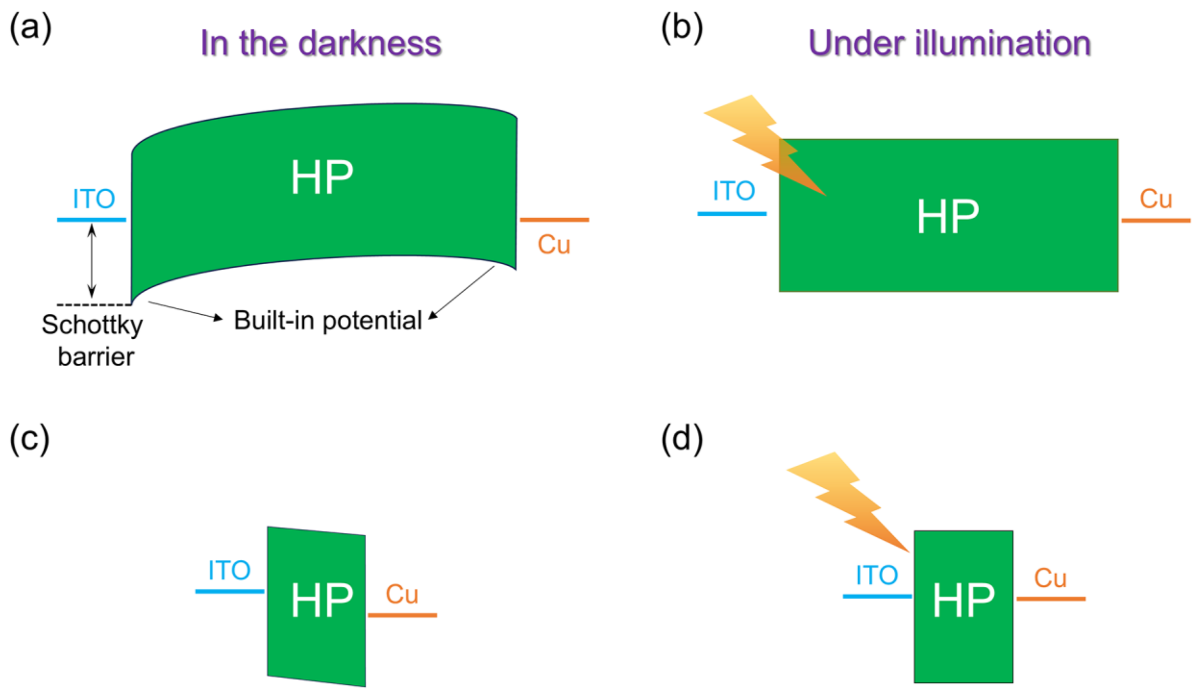


**Fig. S15. Schematic illustrations of the energy landscapes of ITO/$BA_2PbI_4$/Cu devices in the dark and under illumination.** (a) and (b) depict the energy landscapes for the thick $BA_2PbI_4$ film (337 nm), while (c) and (d) represent those for the thin $BA_2PbI_4$ film (29.5 nm). Note that these schematic illustrations focus on the energetics of the HP/metal interfaces and do not reflect the photoinduced built-in potential between the HP sublattice and the BA layer.

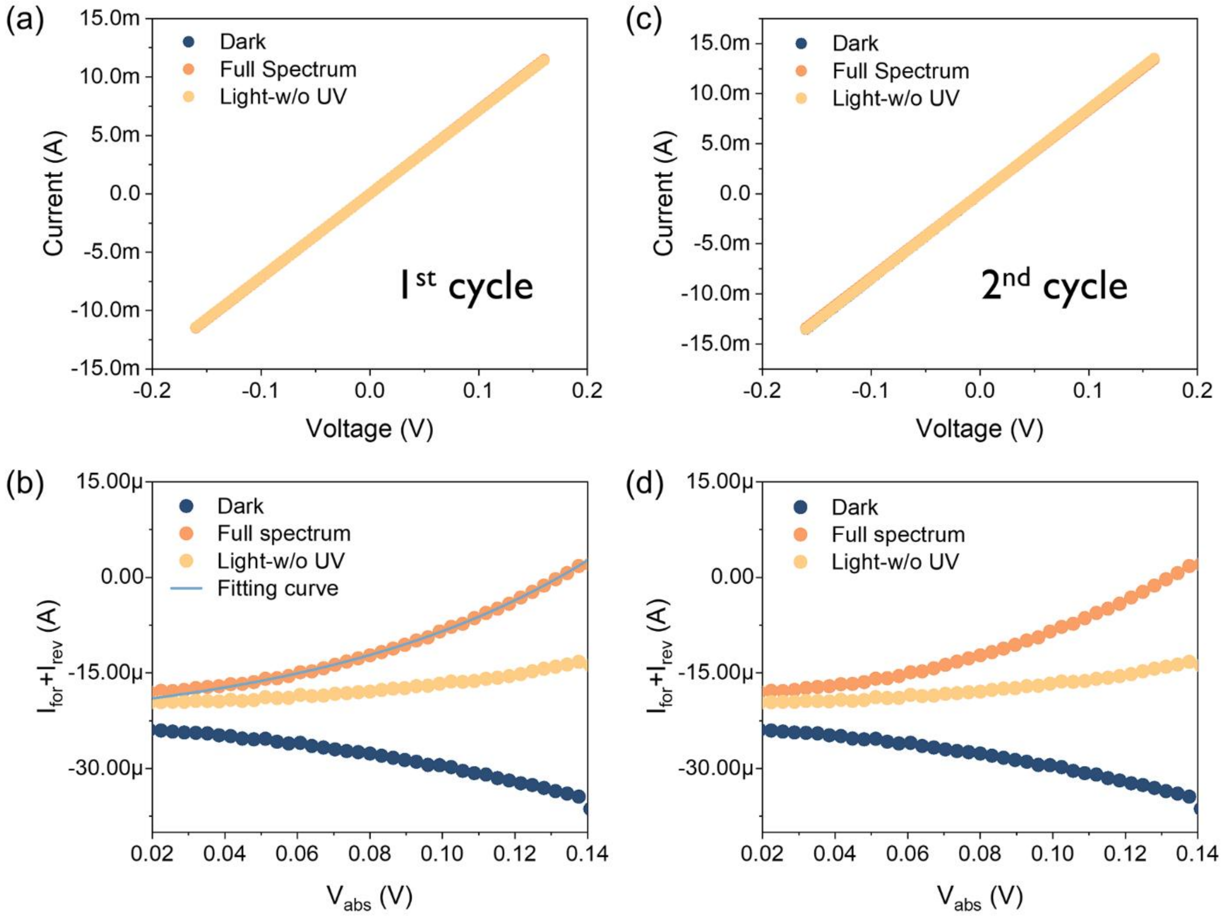


**Fig. S16. Steady-state optoelectrical characterization of the thick $BA_2PbI_4$ film (337 nm) in the device configuration ITO/$BA_2PbI_4$/Cu.** (a) Results from the first round of I-V measurements conducted under three conditions: in the dark, under full-spectrum illumination, and under filtered illumination (photons with wavelengths shorter than 420 nm filtered out). (b) Corresponding ($I_{for}+I_{rev}$) versus $V_{abs}$ characteristics for the three measurement conditions. (c) Results from the second round of I-V measurements and (d) the corresponding ($I_{for}+I_{rev}$) versus $V_{abs}$ characteristics.

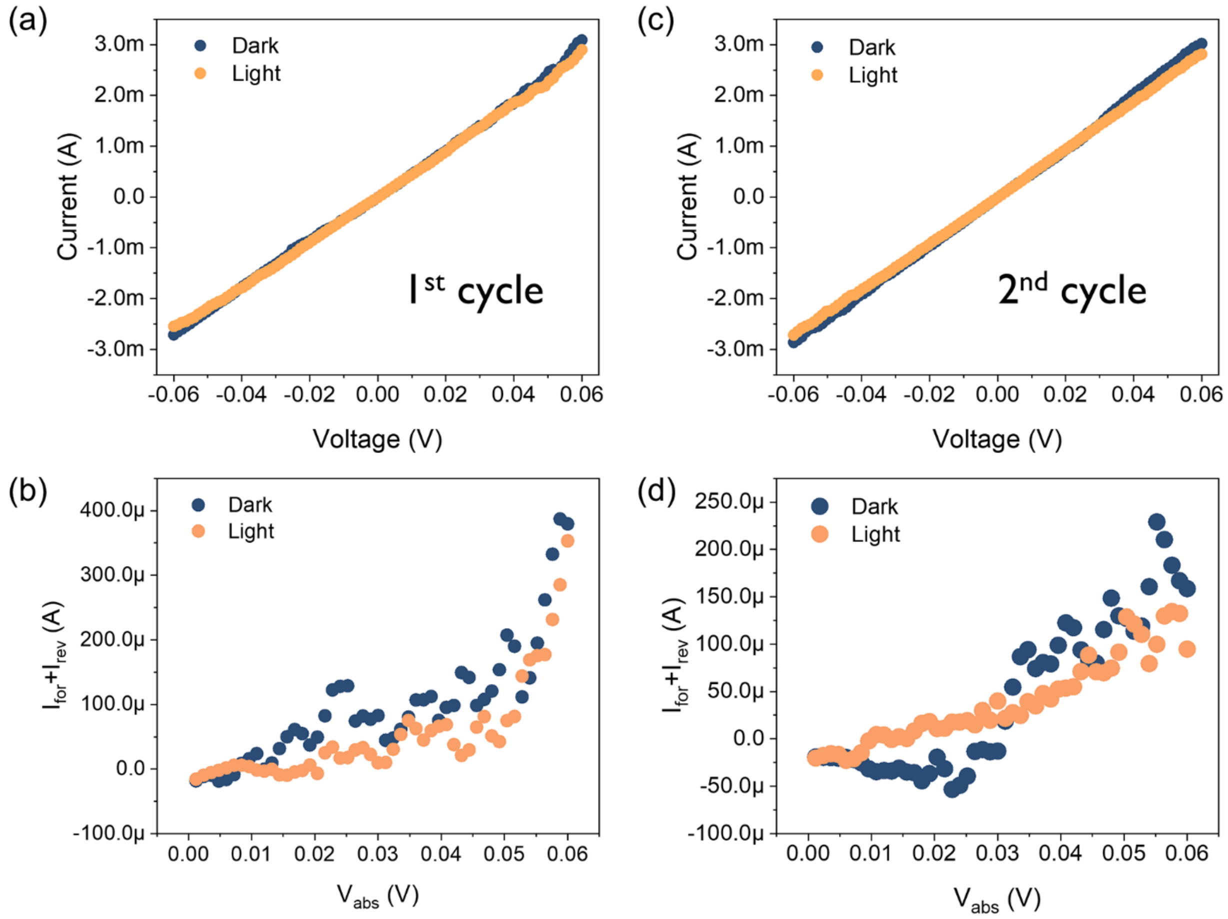


**Fig. S17. Steady-state optoelectrical characterization of the thin $BA_2PbI_4$ film (29.5 nm) in the device configuration ITO/$BA_2PbI_4$/Cu.** (a) Results from the first round of I-V measurements conducted under three conditions: in the dark, under full-spectrum illumination, and under filtered illumination (photons with wavelengths shorter than 420 nm filtered out). (b) Corresponding ($I_{for}$+$I_{rev}$) versus $V_{abs}$ characteristics for the three measurement conditions. (c) Results from the second round of I-V measurements and (d) the corresponding ($I_{for}$+$I_{rev}$) versus $V_{abs}$ characteristics.

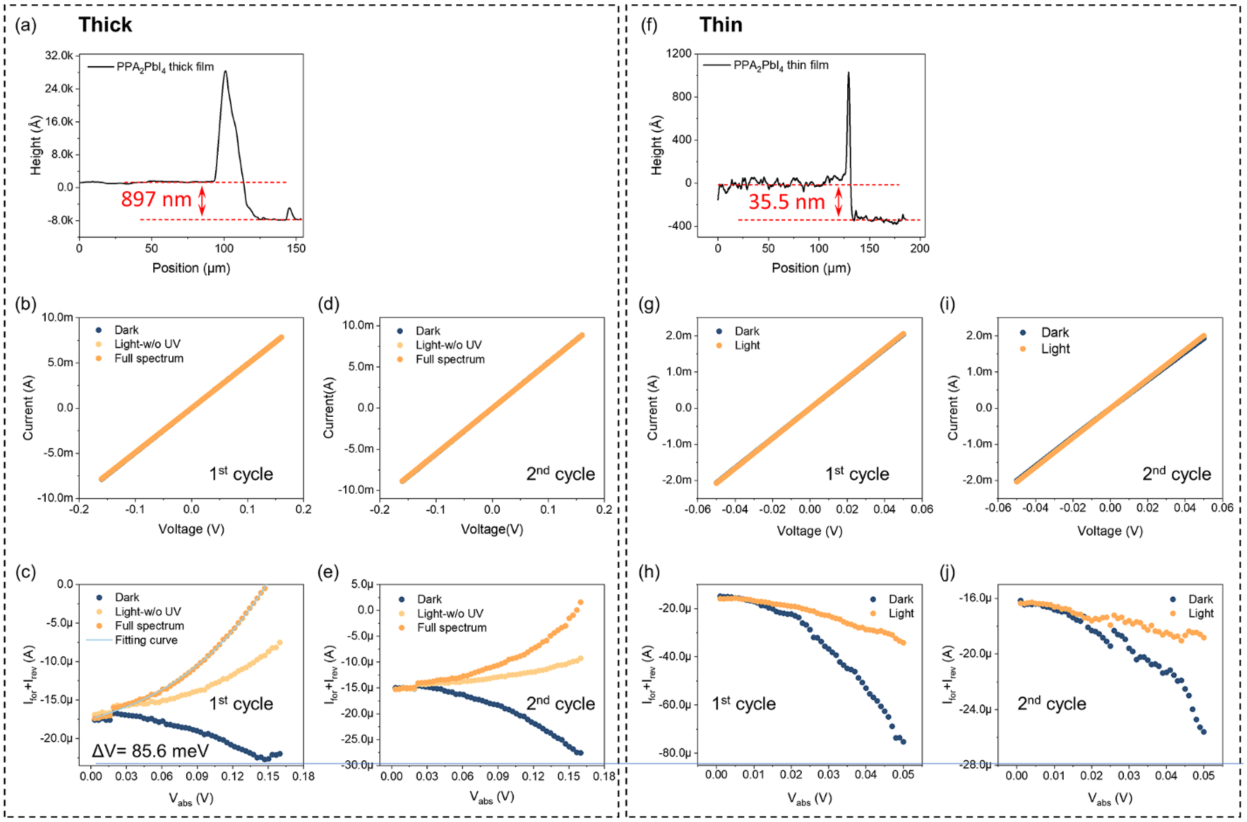


**Fig. S18. Steady-state optoelectrical characterizations of thick and thin $PPA_2PbI_4$ films in the configuration ITO/$PPA_2PbI_4$/Cu.** The left panel presents the double-checked results for the thick film, while the right panel shows the results for the thin film. (a) Thickness characterization of the thick film. (b) I-V curves of the thick film measured for the first time under three conditions: in the dark, under full-spectrum simulated solar illumination, and under filtered illumination (photons with wavelengths shorter than 420 nm filtered out). (c) Corresponding ($I_{for}$+$I_{rev}$) versus $V_{abs}$ characteristics for the three conditions. (d)-(e) Results from the second measurement for verification. (f) Thickness characterization of the thin film. (g) I-V curves of the thin film measured for the first time under two conditions: in the dark and under full-spectrum simulated solar illumination. (h) Corresponding ($I_{for}$+$I_{rev}$) versus $V_{abs}$ characteristics for the two conditions. (i)-(j) Results from the second measurement for verification.

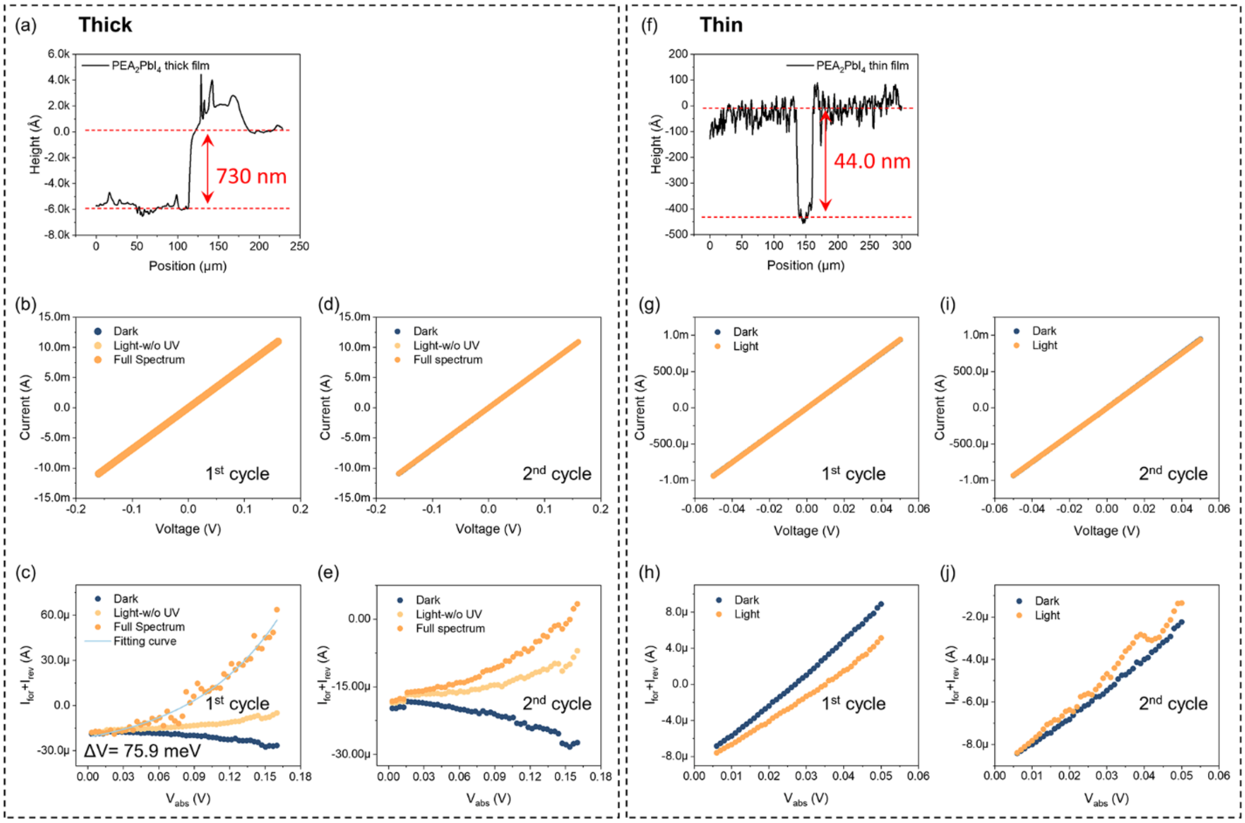


**Fig. S19. Steady-state optoelectrical characterizations of thick and thin $PEA_2PbI_4$ films in the configuration ITO/$PEA_2PbI_4$/Cu.** The left panel presents the double-checked results for the thick film, while the right panel shows the results for the thin film. (a) Thickness characterization of the thick film. (b) I-V curves of the thick film measured for the first time under three conditions: in the dark, under full-spectrum simulated solar illumination, and under filtered illumination (photons with wavelengths shorter than 420 nm filtered out). (c) Corresponding ($I_{for}$+$I_{rev}$) versus $V_{abs}$ characteristics for the three conditions. (d)-(e) Results from the second measurement for verification. (f) Thickness characterization of the thin film. (g) I-V curves of the thin film measured for the first time under two conditions: in the dark and under full-spectrum simulated solar illumination. (h) Corresponding ($I_{for}$+$I_{rev}$) versus $V_{abs}$ characteristics for the two conditions. (i)-(j) Results from the second measurement for verification.

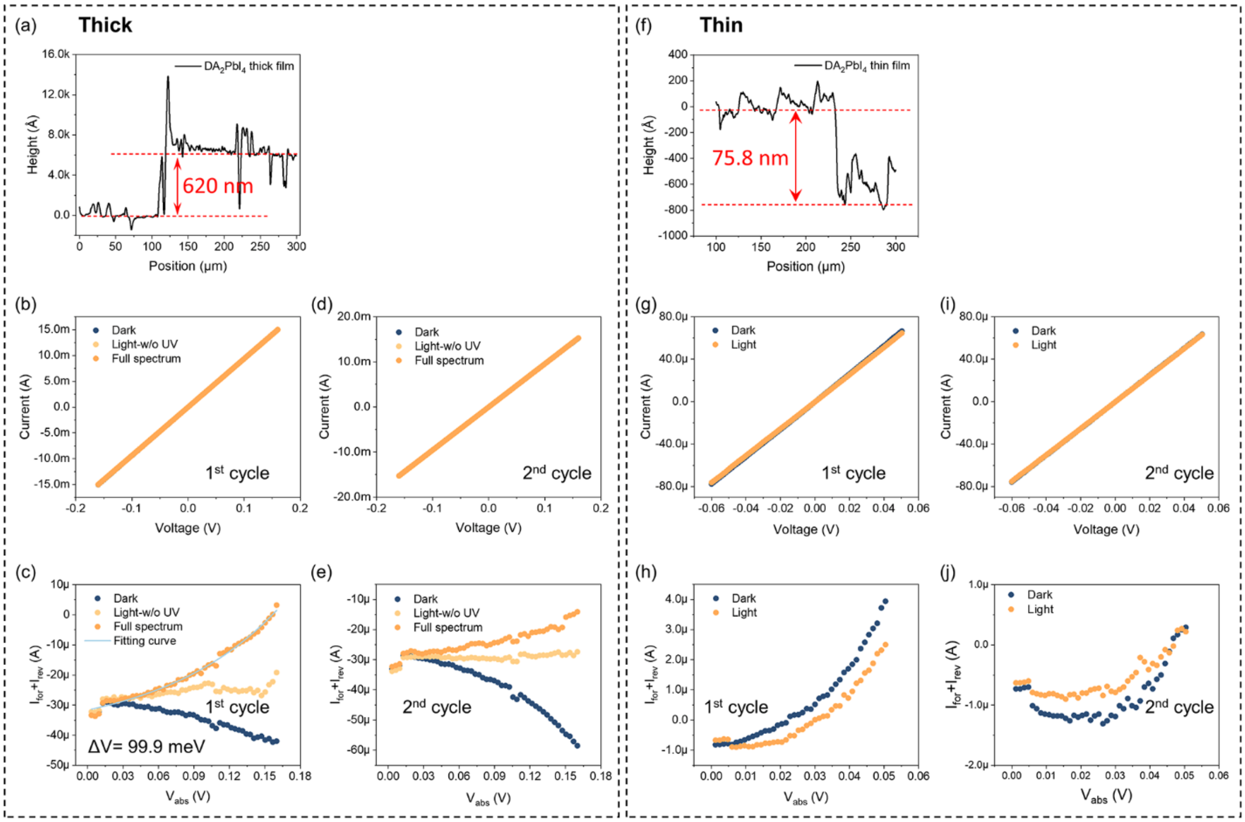


**Fig. S20. Steady-state optoelectrical characterizations of thick and thin $DA_2PbI_4$ films in the configuration ITO/$DA_2PbI_4$/Cu.** The left panel presents the double-checked results for the thick film, while the right panel shows the results for the thin film. (a) Thickness characterization of the thick film. (b) I-V curves of the thick film measured for the first time under three conditions: in the dark, under full-spectrum simulated solar illumination, and under filtered illumination (photons with wavelengths shorter than 420 nm filtered out). (c) Corresponding ($I_{for}+I_{rev}$) versus $V_{abs}$ characteristics for the three conditions. (d)-(e) Results from the second measurement for verification. (f) Thickness characterization of the thin film. (g) I-V curves of the thin film measured for the first time under two conditions: in the dark and under full-spectrum simulated solar illumination. (h) Corresponding ($I_{for}+I_{rev}$) versus $V_{abs}$ characteristics for the two conditions. (i)-(j) Results from the second measurement for verification.

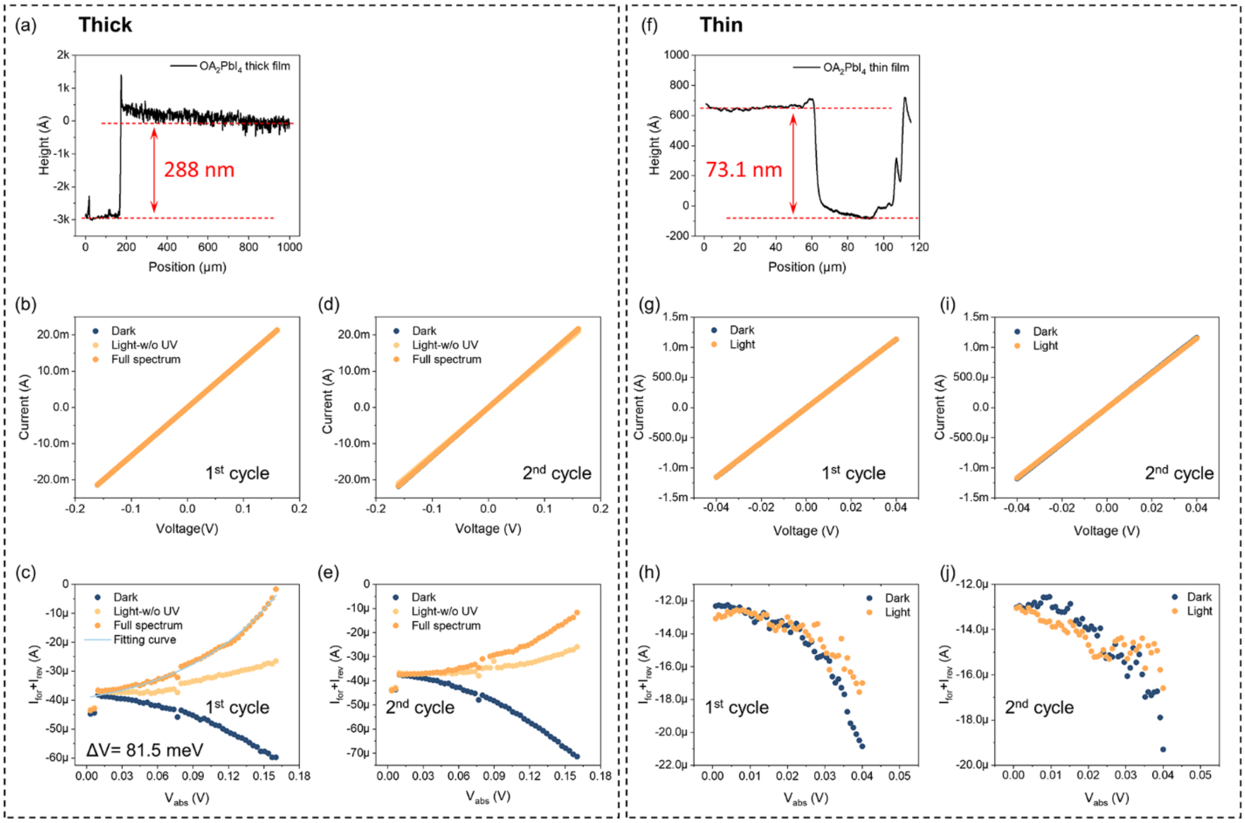


**Fig. S21. Steady-state optoelectrical characterizations of thick and thin $OA_2PbI_4$ films in the configuration ITO/$OA_2PbI_4$/Cu.** The left panel presents the double-checked results for the thick film, while the right panel shows the results for the thin film. (a) Thickness characterization of the thick film. (b) I-V curves of the thick film measured for the first time under three conditions: in the dark, under full-spectrum simulated solar illumination, and under filtered illumination (photons with wavelengths shorter than 420 nm filtered out). (c) Corresponding ($I_{for}$+$I_{rev}$) versus $V_{abs}$ characteristics for the three conditions. (d)-(e) Results from the second measurement for verification. (f) Thickness characterization of the thin film. (g) I-V curves of the thin film measured for the first time under two conditions: in the dark and under full-spectrum simulated solar illumination. (h) Corresponding ($I_{for}$+$I_{rev}$) versus $V_{abs}$ characteristics for the two conditions. (i)-(j) Results from the second measurement for verification.

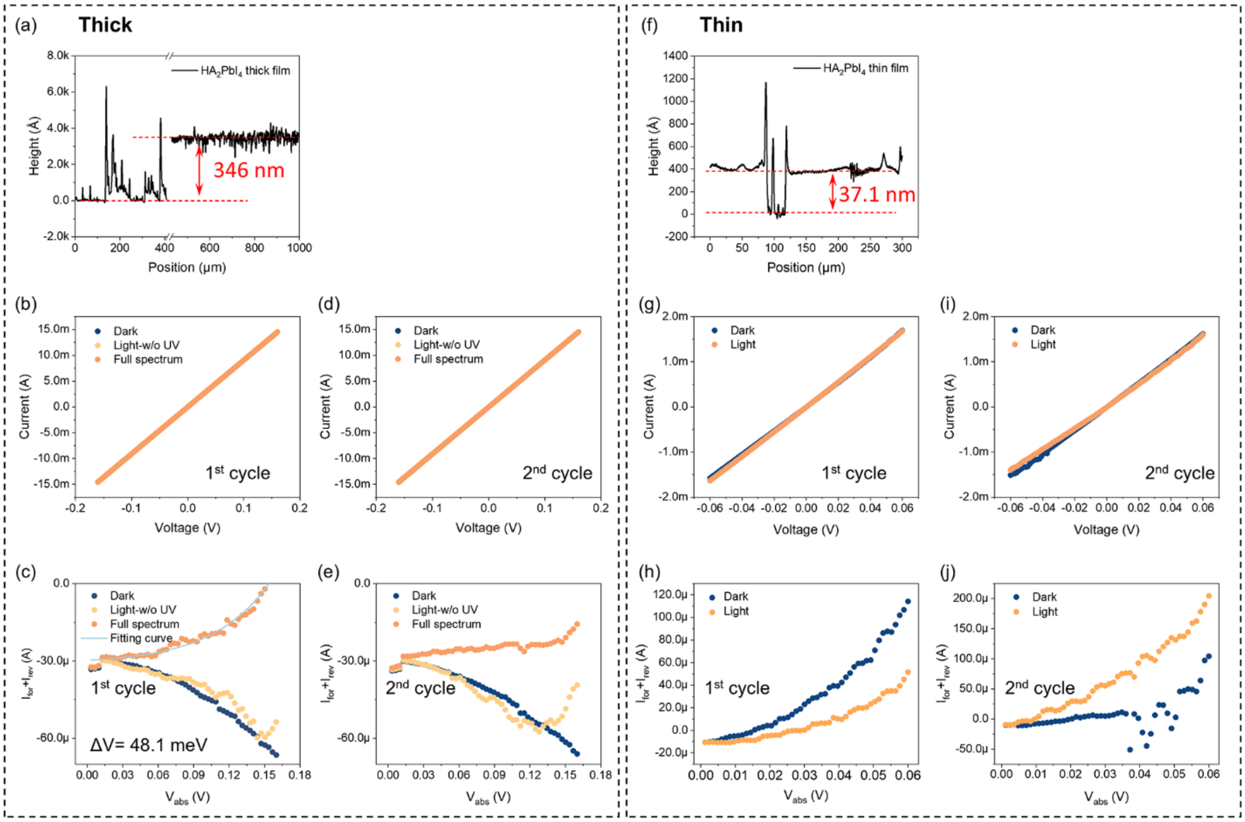


**Fig. S22. Steady-state optoelectrical characterizations of thick and thin $HA_2PbI_4$ films in the configuration ITO/$HA_2PbI_4$/Cu.** The left panel presents the double-checked results for the thick film, while the right panel shows the results for the thin film. (a) Thickness characterization of the thick film. (b) I-V curves of the thick film measured for the first time under three conditions: in the dark, under full-spectrum simulated solar illumination, and under filtered illumination (photons with wavelengths shorter than 420 nm filtered out). (c) Corresponding ($I_{for}$+$I_{rev}$) versus $V_{abs}$ characteristics for the three conditions. (d)-(e) Results from the second measurement for verification. (f) Thickness characterization of the thin film. (g) I-V curves of the thin film measured for the first time under two conditions: in the dark and under full-spectrum simulated solar illumination. (h) Corresponding ($I_{for}$+$I_{rev}$) versus $V_{abs}$ characteristics for the two conditions. (i)-(j) Results from the second measurement for verification.

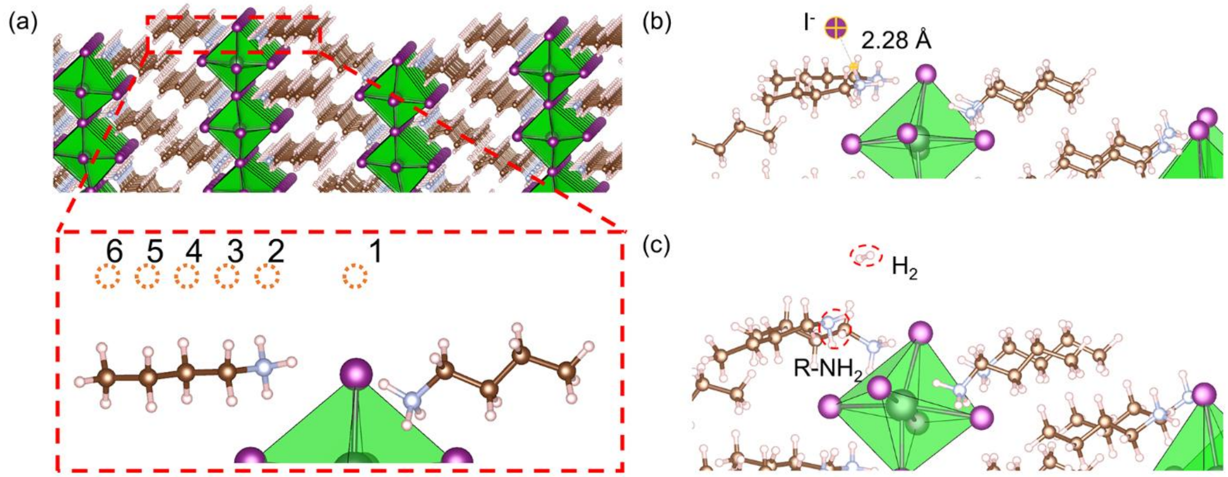


**Fig. S23. DFT results on the photoreaction system.** (a) Simulation of the adsorption sites for $H^+$ and $I^-$ from the HI solution. Site 1 is the iodine ion of lead iodide octahedron; Site 2 is the nitrogen atom of the ammonium group; Sites 3-6 are the carbon atoms in the side alkyl chain. (b) Schematic illustration of the $I^-$ adsorption to the ammonium group. (c) Schematic illustration of the photoreaction of $H^+$, resulting in $H_2$ and deprotonated ligands.

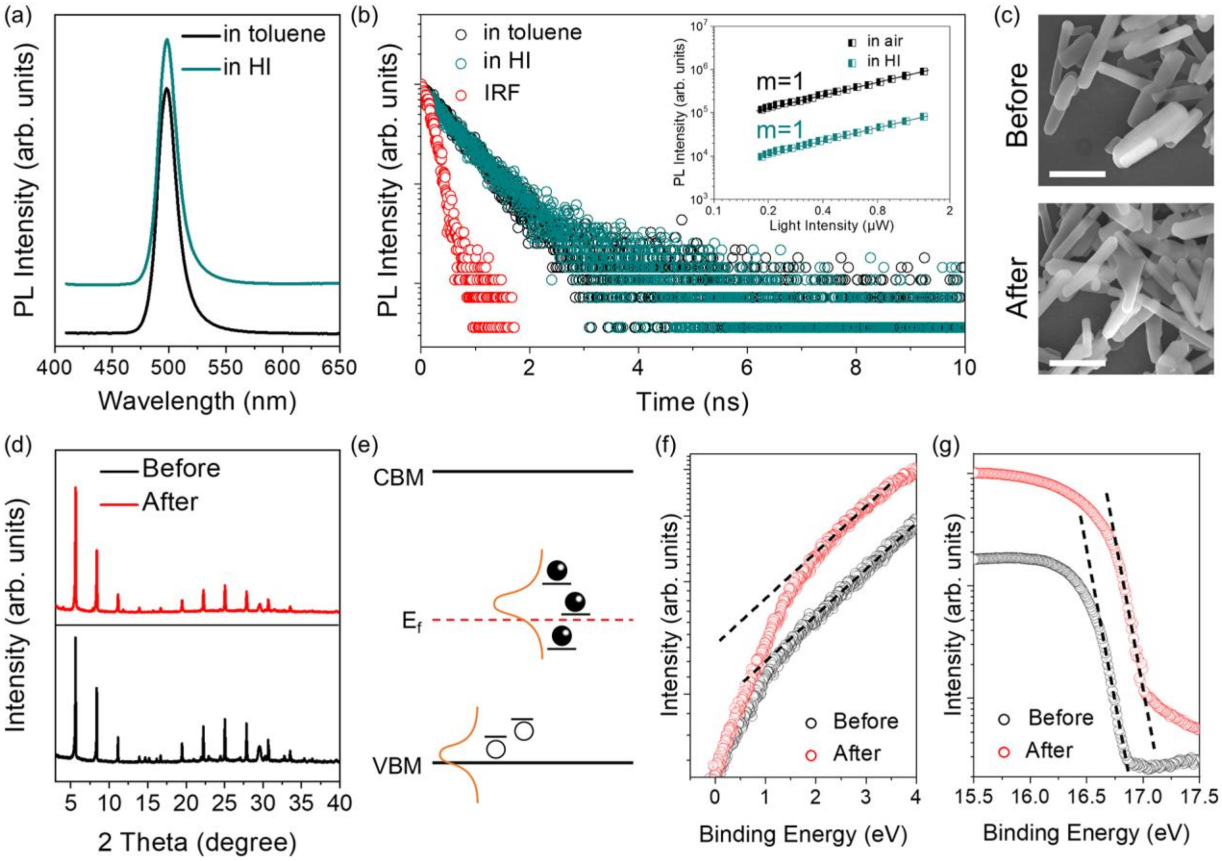


**Fig. S24. Characterizations showing the robustness of $ODA_2PbI_4$ throughout the photoreaction experiment.** (a) Comparative PL spectra of the $ODA_2PbI_4$ nanoplates in toluene and the HI solution, respectively. (b) Comparative TRPL spectra of the $ODA_2PbI_4$ nanoplates in toluene and the HI solution, respectively. IRF denotes instrument response function. Inset: The integrated PL intensity as a function of excitation intensity, measured under ambient air conditions and in the HI solution environment, respectively. m is the power exponent of the relationship $I_{PL} \propto P^m$, where $I_{PL}$ is the intensity of PL, and P is the intensity of excitation. (c) SEM micrographs of the $ODA_2PbI_4$ nanoplates before and after 6 h of $H_2$ evolution in the HI solution. The scale bars are 5 μm. (d) Powder XRD patterns of the $ODA_2PbI_4$ nanoplates before and after 6 h of $H_2$. (e) Schematic illustration of the energy disorders near the Fermi level and the valence band. The orange curves depict the distribution of states. UPS spectra of the $ODA_2PbI_4$ nanosheets before and after the $H_2$ evolution, with zoom-in views of (f) the valence band regions and (g) the secondary electron cut-off regions.

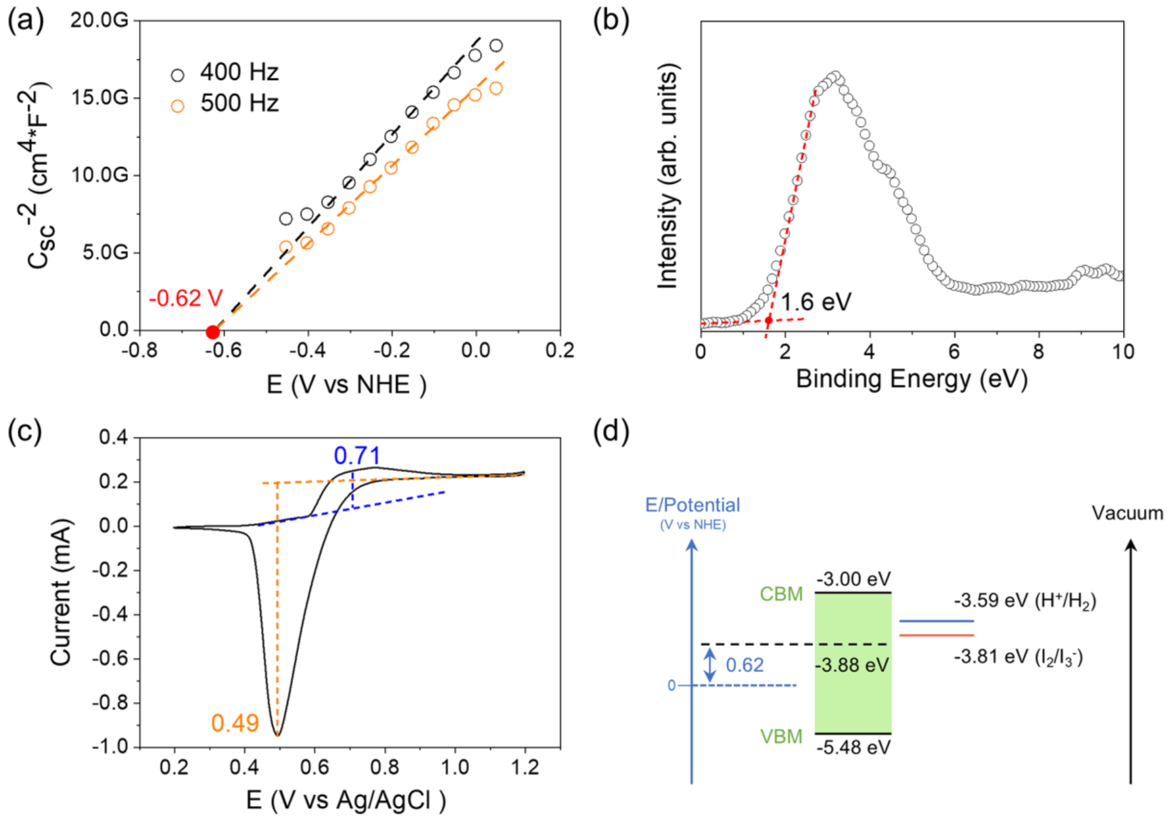


**Fig. S25. Energy landscape of the 2D HP/HI interface that allows for photocatalytic $H_2$ generation.** (a) Mott-Schottky analysis of the 2D HP ($ODA_2PbI_4$) material. (b) XPS valence band spectrum of the 2D HP, revealing an energy gap of 1.6 eV between the Fermi level and the VBM. (c) Capacitance-voltage characteristics of the HI solution. (d) Alignment between the energy band structure of the 2D HP (specifically the inorganic HP component) and the redox potentials of the HI solution, illustrating the favorable energetic driving forces for photocatalytic hydrogen evolution at this heterojunction interface.

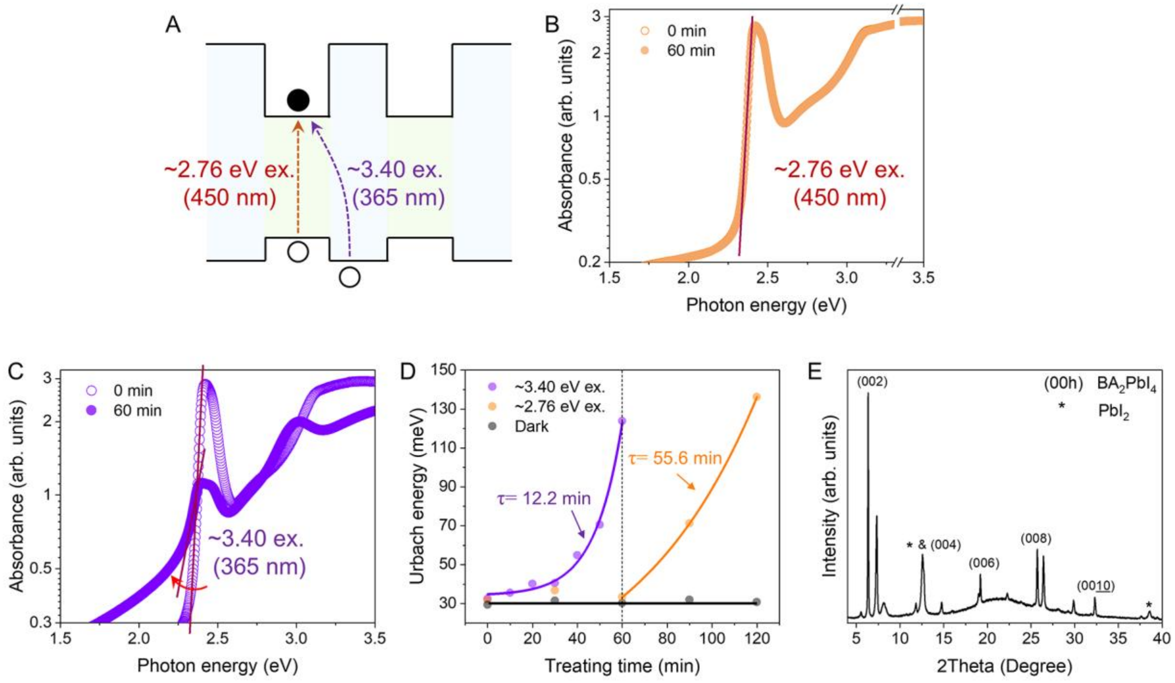


**Fig. S26. Violet/UV light-induced degradation of the 2D HP $BA_2PbI_4$.** (a) Schematic diagram of electron transition dynamics upon monochromatic excitation at ~3.40 eV (365 nm) and ~2.76 eV (450 nm). Comparative absorption spectra of the 2D HP films, measured at 0 minutes and 60 minutes under (b) ~2.76 eV irradiation and (c) ~3.40 eV irradiation, respectively. Both irradiation intensities were maintained at 20 mW $cm^{-2}$, corresponding to absorbed photon fluxes of $3.40 \times 10^{16}$ photons $s^{-1}$ $cm^{-2}$ for the ~3.40 eV excitation and $4.22 \times 10^{16}$ photons $s^{-1}$ $cm^{-2}$ for the ~2.76 eV excitation. The measurements were carried out under ambient air conditions. (D) Comparison of the Urbach energy evolution in the 2D HP film under different illumination conditions, including excitation at ~3.40 eV (365 nm), ~2.76 eV (450 nm), and in the dark. (e) The XRD pattern of the $BA_2PbI_4$ film after being irradiated by the ~3.40 eV (365 nm) illumination for 60 minutes.

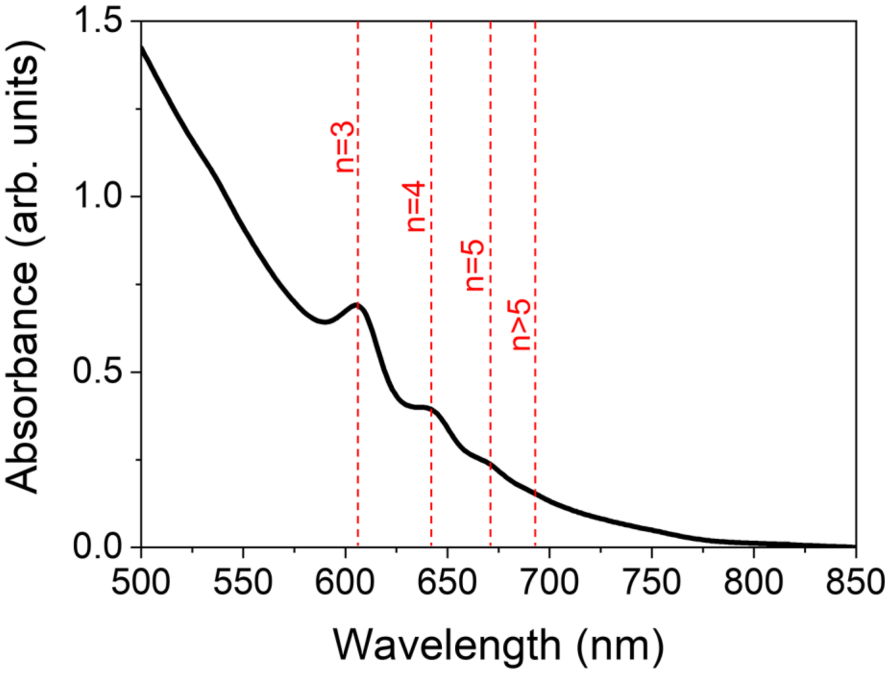


**Fig. S27. Absorption spectrum of the $BA_2MA_4Pb_5I_{16}$ film (MA denotes methyl ammonium).** Steady-state absorption spectrum of the multiphasic quasi-2D HP film with a nominal <n> value of 5. The general chemical formula of the film is $BA_2MA_4Pb_5I_{16}$.

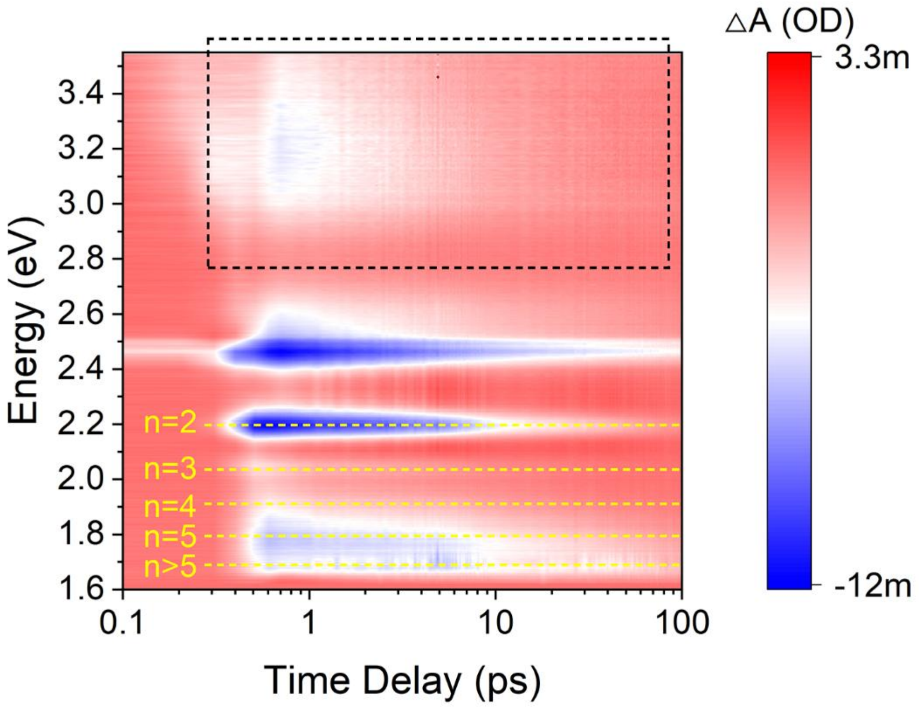


**Fig. S28. The TA spectrum of the $PPA_2MA_4Pb_5I_{16}$ film.** Pseudocolor ultrafast TA spectrum of the quasi-2D HP $PPA_2MA_4Pb_5I_{16}$ film with a nominal <n> value of 5. The excitation energy is 2.48 eV (500 nm, 1 kHz, 100 fs).

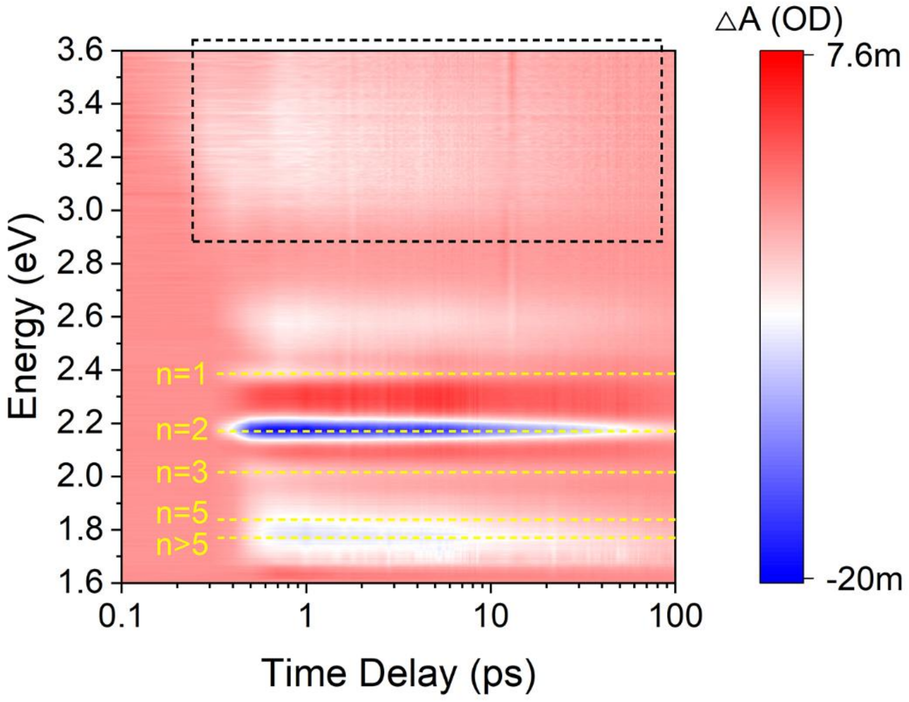


**Fig. S29. The TA spectrum of the $PEA_2MA_4Pb_5I_{16}$ film.** Pseudocolor ultrafast TA spectrum of the quasi-2D HP $PEA_2MA_4Pb_5I_{16}$ film with a nominal <n> value of 5. The excitation energy is 2.48 eV (500 nm, 1 kHz, 100 fs).

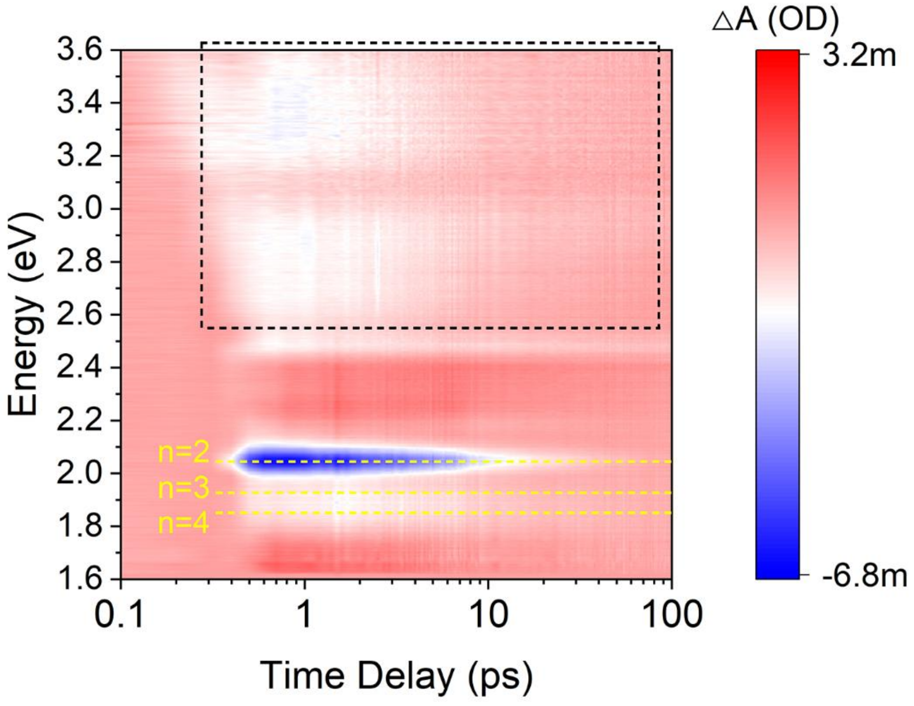


**Fig. S30. The TA spectrum of the $OA_2MA_4Pb_5I_{16}$ film.** Pseudocolor ultrafast TA spectrum of the quasi-2D HP $OA_2MA_4Pb_5I_{16}$ film with a nominal <n> value of 5. The excitation energy is 2.48 eV (500 nm, 1 kHz, 100 fs).

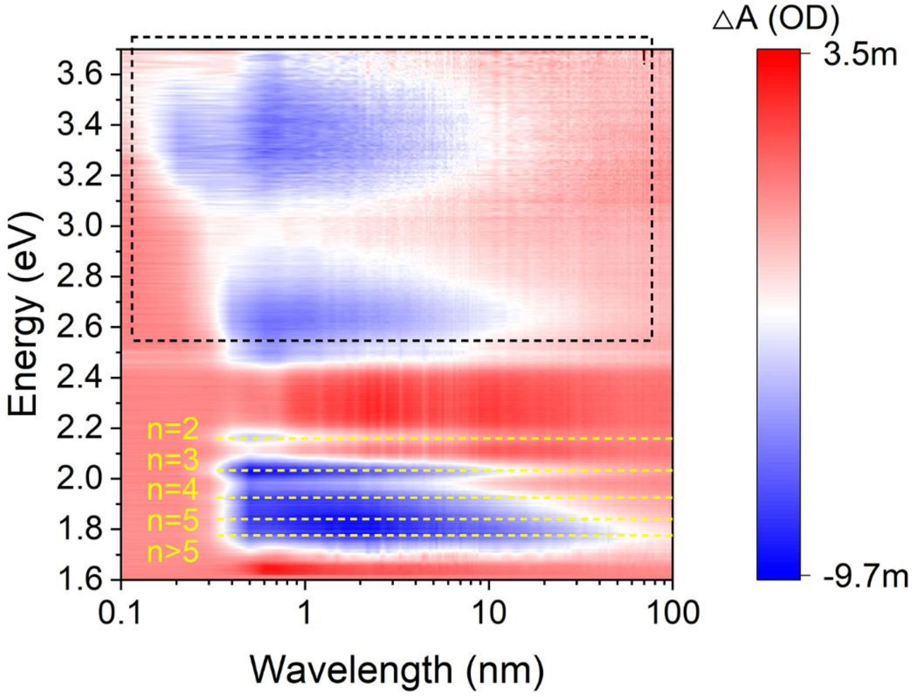


**Fig. S31. The TA spectrum of the $HA_2MA_4Pb_5I_{16}$ film.** Pseudocolor ultrafast TA spectrum of the quasi-2D HP $HA_2MA_4Pb_5I_{16}$ film with a nominal <n> value of 5. The excitation energy is 2.48 eV (500 nm, 1 kHz, 100 fs).

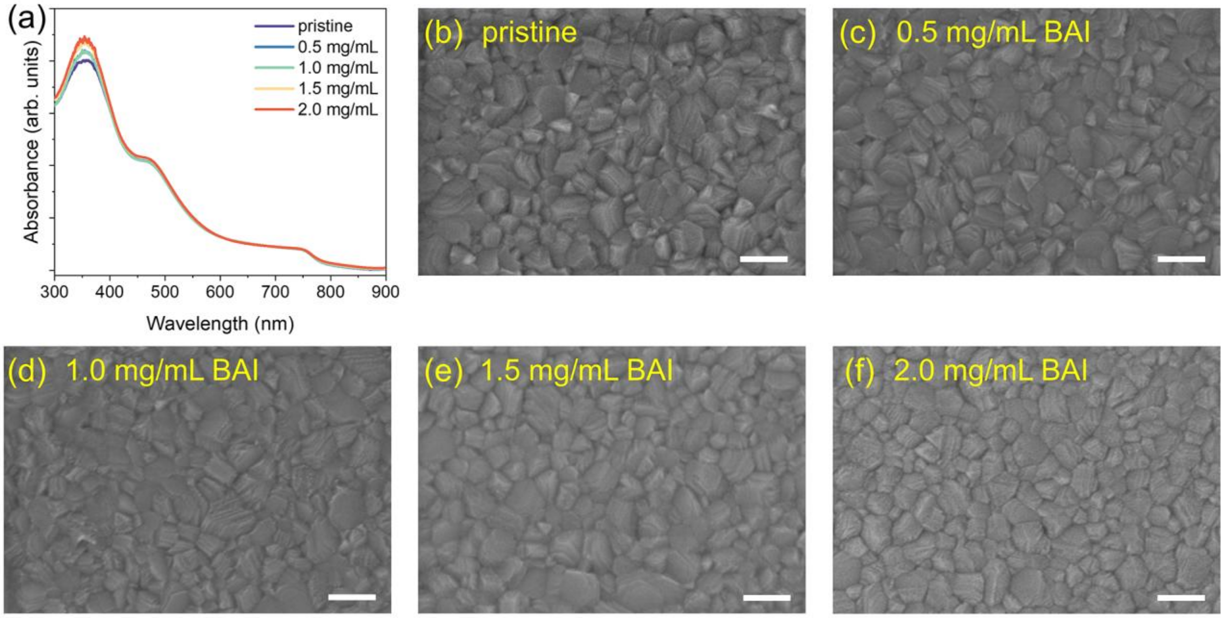


**Fig. S32. The absorption change and morphology change of $MAPbI_3$ films upon the surface treatment.** (a) Steady-state absorption spectra of 3D HP ($MAPbI_3$) films treated by BAI solutions with a gradient of concentrations on the surface. (b) The initial morphology of the untreated 3D $MAPbI_3$ film. (c)-(f) the corresponding SEM micrographs of the 3D $MAPbI_3$ film after being treated by BAI solutions with different concentrations. The scale bars are 200 nm. The steady-state absorption spectra characterization and the SEM characterization show no formation of 2D HP phases after the BAI treatment.

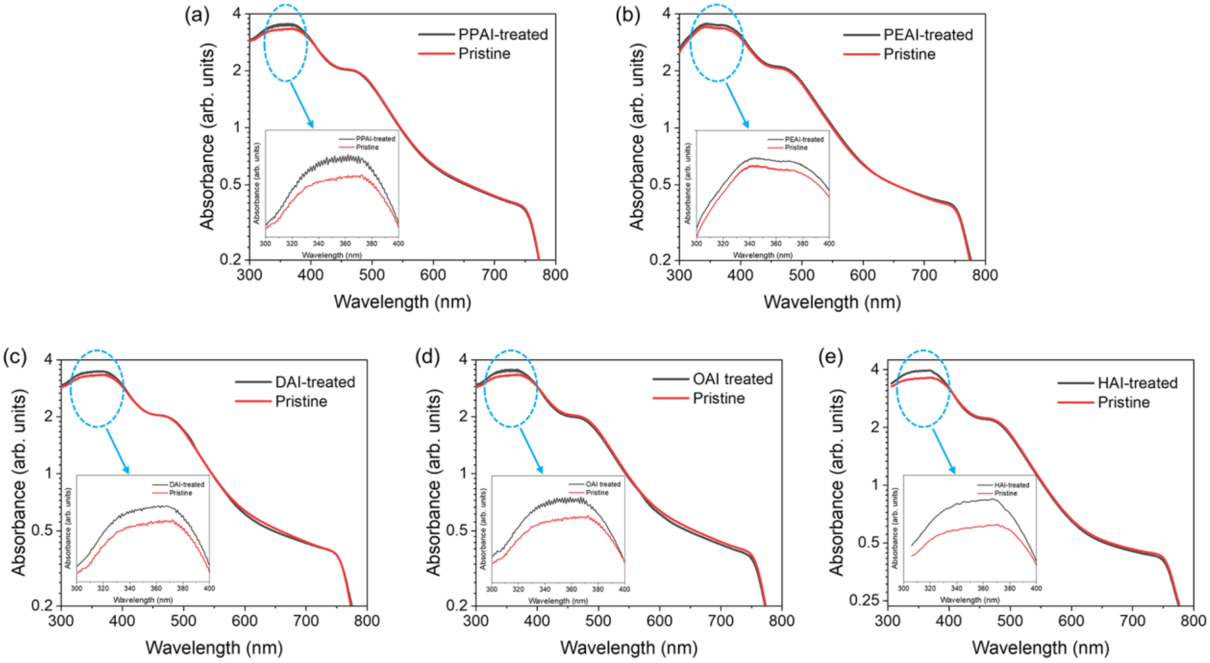


**Fig. S33. The steady-state absorption spectra of 3D HP $MAPbI_3$ before and after surface treatments with various ammonium ligands.** These ammonium ligands include (a) PPA (in the form of PPAI), (b) PEA (in the form of PEAI), (c) DA (in the form of DAI), (d) OA (in the form of OAI), and (e) HA (in the form of HAI). Insets: Zoom-in regions of absorption spectra in the range of 300-400 nm.

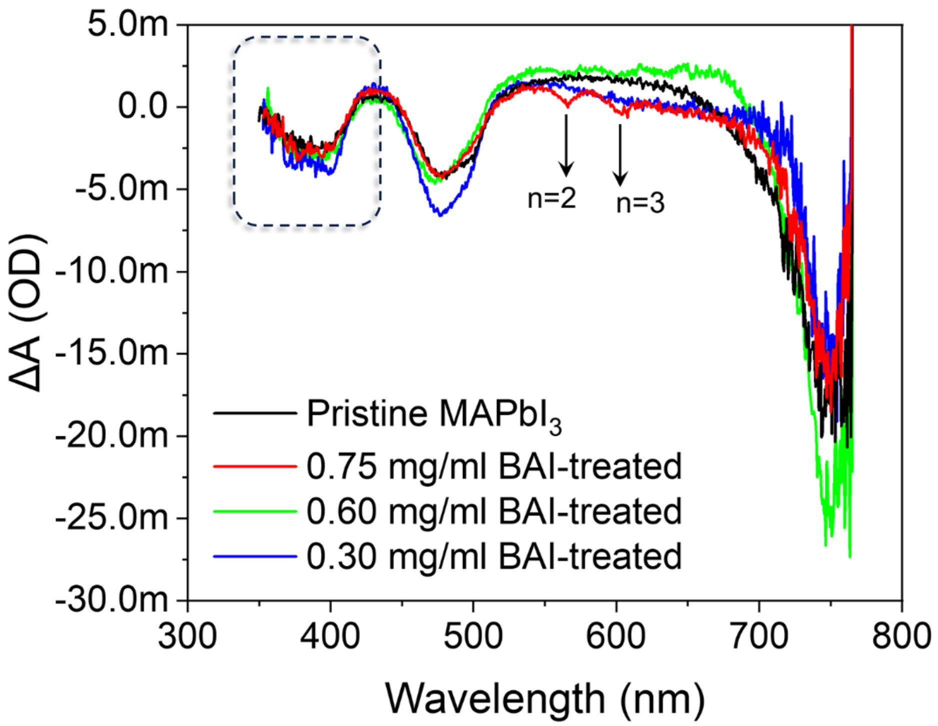


**Fig. S34. TA spectra of 3D $MAPbI_3$ HP films treated with BAI.** The surface treatment with ammonium ligands can easily cause the formation of 2D HPs, which complicates the interpretation of the PB signals. The formation of 2D HPs is even exacerbated in the 3D HP film for the TA measurement, as the film needs to be very thin, so the 3D-to-2D conversion is easier to occur. It was not until the concentration of BAI was reduced to 0.3 mg/ml that the formation of 2D HPs was prevented. However, $MAPbI_3$ itself exhibits high-energy absorption signals, one of which broadly distributes in the ultraviolet region and overlaps with the ammonium-HP interaction signal. This overlap makes it challenging to resolve the ammonium-HP interaction signal in ammonium-passivated 3D HPs using TA spectrum measurements.

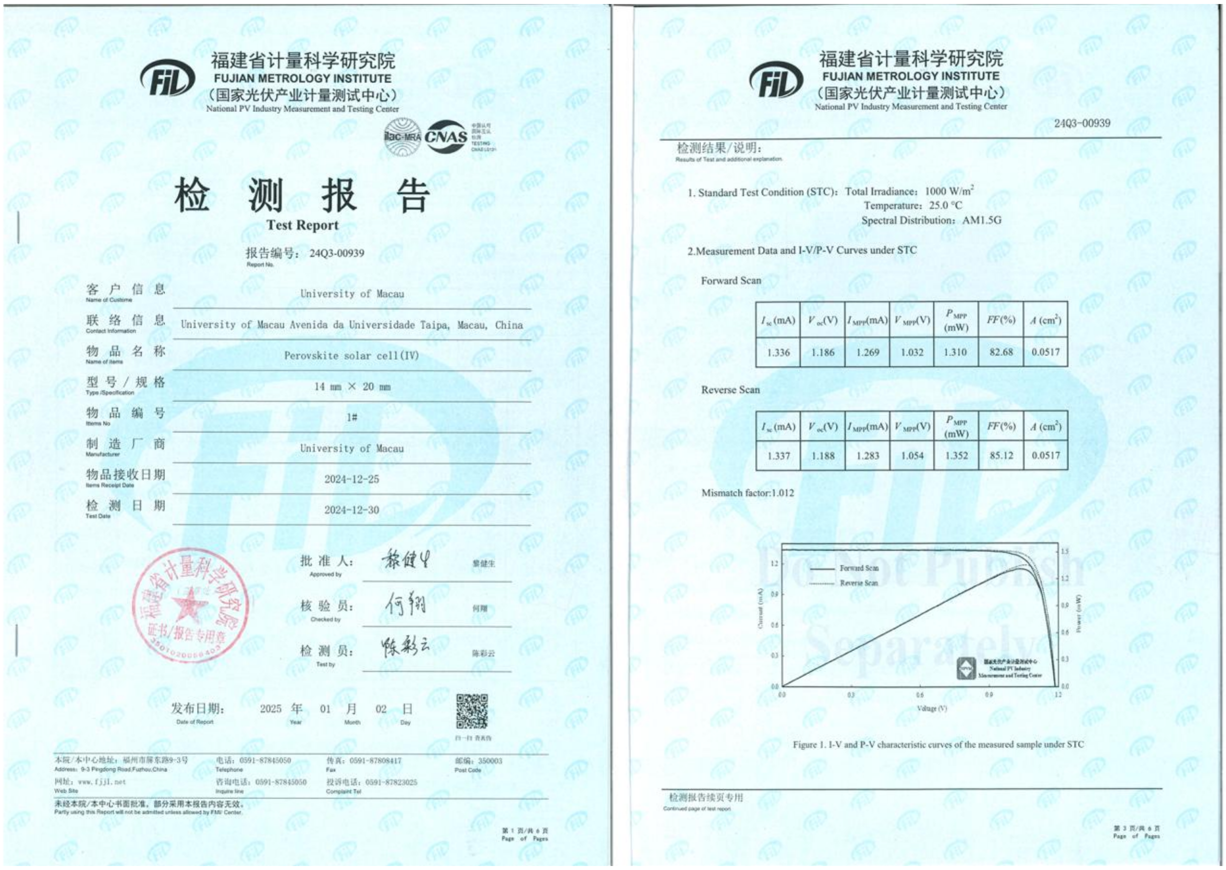

福建省计量科学研究院
FUJIAN METROLOGY INSTITUTE
(国家光伏产业计量测试中心)
National PV Industry Measurement and Testing Center

检 测 报 告
Test Report

报告编号: 24Q3-00939
Report No.

| | |
|---|---|
| 客户信息 Name of Custome | University of Macau |
| 联络信息 Contact Information | University of Macau Avenida da Universidade Taipa, Macau, China |
| 物品名称 Name of items | Perovskite solar cell(IV) |
| 型号/规格 Type /Specification | 14 mm × 20 mm |
| 物品编号 Items No | 1# |
| 制造厂商 Manufacturer | University of Macau |
| 物品接收日期 Items Receipt Date | 2024-12-25 |
| 检测日期 Test Date | 2024-12-30 |

批准人: Approved by 黎健生
核验员: Checked by 何翔
检测员: Test by 陈彩云

发布日期: 2025 年 01 月 02 日
Date of Report Year Month Day

福建省计量科学研究院
FUJIAN METROLOGY INSTITUTE
(国家光伏产业计量测试中心)
National PV Industry Measurement and Testing Center

24Q3-00939

检测结果/说明:
Results of Test and additional explanation.

1. Standard Test Condition (STC): Total Irradiance: 1000 W/m$^2$
Temperature: 25.0 °C
Spectral Distribution: AM1.5G

2.Measurement Data and I-V/P-V Curves under STC

Forward Scan

| $I_{sc}$ (mA) | $V_{oc}$ (V) | $I_{MPP}$ (mA) | $V_{MPP}$ (V) | $P_{MPP}$ (mW) | $FF$ (%) | $A$ (cm$^2$) |
|---|---|---|---|---|---|---|
| 1.336 | 1.186 | 1.269 | 1.032 | 1.310 | 82.68 | 0.0517 |

Reverse Scan

| $I_{sc}$ (mA) | $V_{oc}$ (V) | $I_{MPP}$ (mA) | $V_{MPP}$ (V) | $P_{MPP}$ (mW) | $FF$ (%) | $A$ (cm$^2$) |
|---|---|---|---|---|---|---|
| 1.337 | 1.188 | 1.283 | 1.054 | 1.352 | 85.12 | 0.0517 |

Mismatch factor:1.012



Figure 1. I-V and P-V characteristic curves of the measured sample under STC

检测报告续页专用
Continued page of test report

**Fig. S35. The solar cell certification report.** The certified performance report of the ammonium ligand-passivated solar cell.

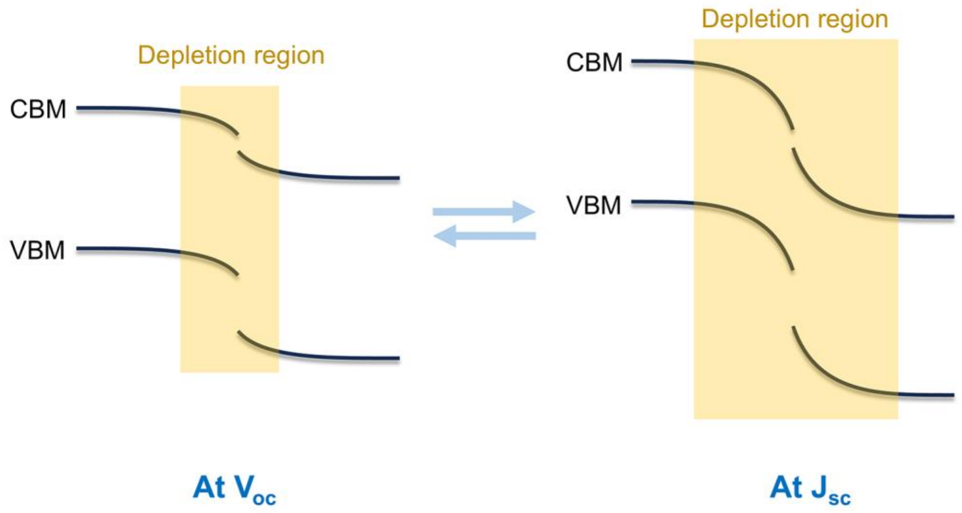


**Figure S36. Schematic diagram of the dynamic energy band bending for a heterojunction during I-V scans.** When at $V_{oc}$, the built-in potential is mitigated by the applied bias voltage to the extent that the drift current equals the diffusion current; when at $J_{sc}$, no external bias voltage is applied, and the built-in electric field is at its maximum.

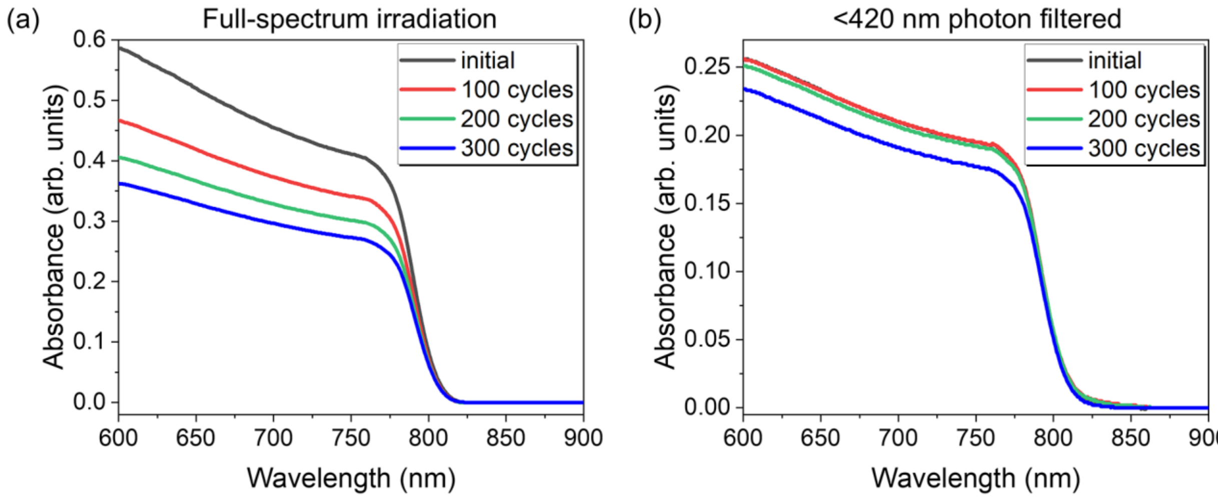


**Fig. S37. The absorption spectrum evolution of the device subjected to the fatigue test.** Evolutions of the baseline-corrected absorption spectra of solar cell devices subjected to (a) the non-filtered, full-spectrum illumination and (b) the filtered illumination, respectively, during the fatigue test. Despite a high reflectivity caused by the Ag electrodes, the absorption spectra still allow us to gain a glimpse into the degradation of the light-absorbing quality of the perovskite layer, which reflects its degradation during the fatigue test.

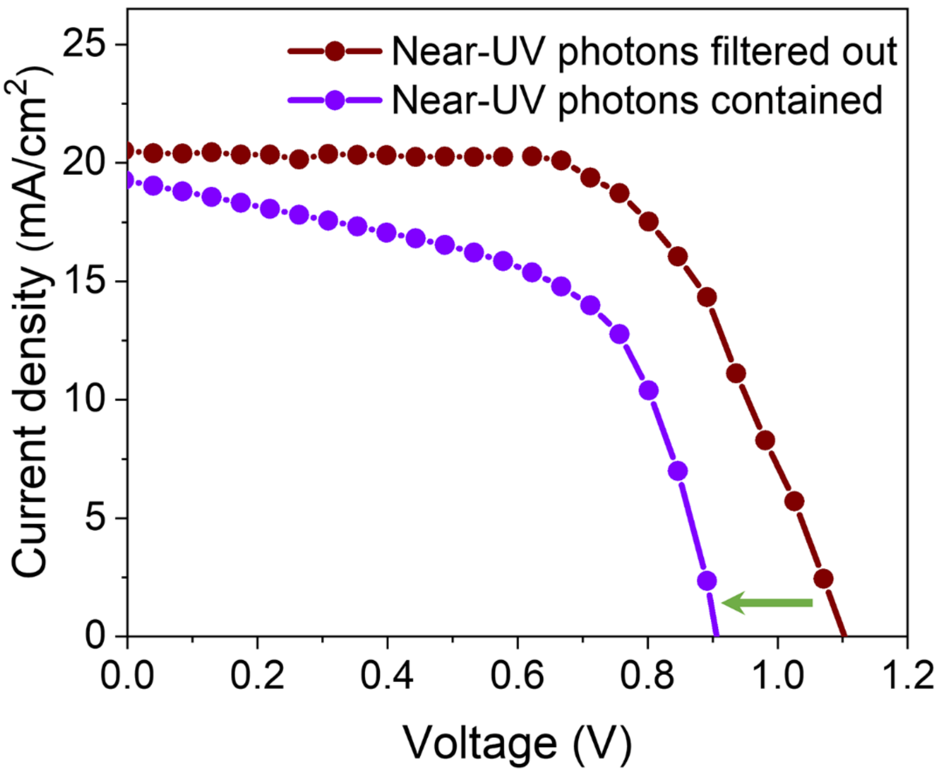


**Fig. S38. Comparative residual I-V characteristics of the HP solar cell after being illuminated by two sources.** One was filtered simulated solar illumination, with photons of wavelength shorter than 420 nm filtered out. The other is a full-spectrum simulated solar illumination source retaining the violet/UV photons. Both illumination tests were carried out in a dry airflow environment.

**Table S1. Summary of device performances for the two solar cells in fig. S38***

| | $V_{oc}$ (V) | FF (%) | $J_{sc}$ (mA/cm$^2$) | PCE (%) |
|---|---|---|---|---|
| filtered | 1.103 | 64.72 | 20.50 | 14.64 |
| unfiltered | 0.906 | 57.15 | 19.23 | 9.96 |

*Note: $V_{oc}$ stands for open-circuit voltage, FF for fill factor, $J_{sc}$ for short-circuit current, and PCE for photoelectric conversion efficiency.

## Supplementary References


1.
2. Wang, H. et al. Mechanistic understanding of efficient photocatalytic $H_2$ evolution on two-dimensional layered lead iodide hybrid perovskites. *Angew. Chem., Int. Ed.* **60,** 7376-7381 (2021).
3. Park, S. et al. Photocatalytic hydrogen generation from hydriodic acid using methylammonium lead iodide in dynamic equilibrium with aqueous solution. *Nat. Energy* **2,** 16185 (2016).
4. Wu, Y. et al. Composite of $CH_3NH_3PbI_3$ with reduced graphene oxide as a highly efficient and stable visible-light photocatalyst for hydrogen evolution in aqueous HI solution. *Adv. Mater.* **30,** 1704342 (2018).
5. Ledinsky, M. et al. Temperature dependence of the Urbach energy in lead iodide perovskites. *J. Phys. Chem. Lett.* **10,** 1368-1373 (2019).
6. Na Quan, L. et al. Edge stabilization in reduced-dimensional perovskites. *Nat. Commun.* **11,** 170 (2020).
7. Farooq, A. et al. Spectral dependence of degradation under ultraviolet light in perovskite solar cells. *ACS Appl. Mater. Interfaces* **10,** 21985-21990 (2018).
8. Wang, Y. et al. Tautomeric molecule acts as a "sunscreen" for metal halide perovskite solar cells. *Angew Chem. Int. Ed. Engl.* **60,** 8673-8677 (2021).
9. Nie, W. et al. Light-activated photocurrent degradation and self-healing in perovskite solar cells. *Nat. Commun.* **7,** 11574 (2016).
10. Khenkin, M. V. et al. Dynamics of photoinduced degradation of perovskite photovoltaics: from reversible to irreversible processes. *ACS Appl. Energy Mater.* **1,** 799-806 (2018).
11. Motti, S. G. et al. Controlling competing photochemical reactions stabilizes perovskite solar cells. *Nat. Photonics* **13,** 532-539 (2019).
12. Mosconi, E., Meggiolaro, D., Snaith, H. J., Stranks, S. D. & De Angelis, F. Light-induced annihilation of Frenkel defects in organo-lead halide perovskites. *Energy Environ. Sci.* **9,** 3180-3187 (2016).
13. Liu, T. et al. Efficient perovskite solar modules enabled by a UV-stable and high-conductivity hole transport material. *Sci. Adv.* **11,** eadu3493 (2025).
14. Fei, C. et al. Strong-bonding hole-transport layers reduce ultraviolet degradation of perovskite solar cells. *Science* **384,** 1126-1134 (2024).
15. Sutter-Fella, C. M. et al. Cation-dependent light-induced halide demixing in hybrid organic-inorganic perovskites. *Nano Lett.* **18,** 3473-3480 (2018).
16. Magill, B. A. et al. Probe of the excitonic transitions and lifetimes in quasi-2D organic–inorganic halide perovskites. *AIP Adv.* **12,** 015114 (2022).
17. Wang, Y. P., Sun, Y. Y., Zhang, S. B., Lu, T. M. & Shi, J. Band gap engineering of a soft inorganic compound $PbI_2$ by incommensurate van der Waals epitaxy. *Appl. Phys. Lett.* **108,** (2016).
