# Supplementary material for: Violet/ultraviolet light-induced depassivation in halide perovskite solar cells
Source: Nat Commun. 2025 Dec 17;16:11409. doi: 10.1038/s41467-025-66227-4 (PMC12739151; doi:10.1038/s41467-025-66227-4)
Supplement: Supplementary file 2 — Reporting Summary [file 41467_2025_66227_MOESM2_ESM.pdf]

nature portfolio

Corresponding author(s): Prof. Xing, Guichuan

Solar Cells Reporting Summary

Nature Portfolio wishes to improve the reproducibility of the work that we publish. This form is intended for publication with all accepted papers reporting the characterization of photovoltaic devices and provides structure for consistency and transparency in reporting. Some list items might not apply to an individual manuscript, but all fields must be completed for clarity.

For further information on Nature Research policies, including our data availability policy, see Authors & Referees.

Experimental design

Please check the following details are reported in the manuscript, and provide a brief description or explanation where applicable.

1. Dimensions

Area of the tested solar cells

- [x] Yes
- [ ] No

The active area is 0.0517 cm² (both in our lab and in the third-party institution). The information is provided in the certification report in Fig. S35 and will be expanded in further revisions.

Explain why this information is not reported/not relevant.

Method used to determine the device area

- [ ] Yes
- [x] No

Provide a description of the method and state where this information can be found in the text.

In our lab, the active area is determined by the overlap between the top and the bottom electrodes; in the third-party certification, it is determined by a mask. The information will be supplemented in further revisions.

2. Current-voltage characterization

Current density-voltage (J-V) plots in both forward and backward direction

- [x] Yes
- [ ] No

Fig. S35 (the certification report)

Voltage scan conditions

- [ ] Yes
- [x] No

Provide a description of the measurement conditions (e.g. scan direction, speed, dwell times).

In Figure 4d, the I-V characteristics were obtained from a reverse scan. The pre-sweep delay was 0.1 s, and the dwell time was 10 ms. The information will be supplemented in further revisions.

Test environment

- [ ] Yes
- [x] No

Provide a description of the test conditions (e.g. characterization temperature, atmosphere, humidity).

The J-V scan was measured in a N2-filled glovebox at room temperature. The information will be supplemented in further revisions.

Protocol for preconditioning of the device before its characterization

- [ ] Yes
- [x] No

Provide a description of the protocol.

There is no preconditioning of the solar cell devices before the J-V characterization. The information will be supplemented in further revisions.

Stability of the J-V characteristic

- [x] Yes
- [ ] No

To serve the scientific claim of this work, we carried out a fatigue J-V test of the device. The details can be found in the text relevant to Figure 4E.

Explain why this information is not reported/not relevant.

3. Hysteresis or any other unusual behaviour

Description of the unusual behaviour observed during the characterization

- [x] Yes
- [ ] No

The hysteresis information can be found in Fig. S35 (the certification report).

Explain why this information is not reported/not relevant.

Related experimental data

- [ ] Yes
- [x] No

Provide a description of the related experimental data.

Hysteresis behaviour is irrelevant to the core scientific report of this work.

4. Efficiency

External quantum efficiency (EQE) or incident photons to current efficiency (IPCE)

☐ Yes
☒ No

Provide a description of the technique used.

The EQE characterization is irrelevant to the core scientific report of this work.

A comparison between the integrated response under the standard reference spectrum and the response measure under the simulator

☐ Yes
☒ No

State where this information can be found in the text.

The efficiency is measured not only in our lab but also in an authoritative third-party institution, making the efficiency data reliable.

For tandem solar cells, the bias illumination and bias voltage used for each subcell

☐ Yes
☒ No

Provide a description of the measurement conditions.

There is no tandem solar cell in our report.

5. Calibration

Light source and reference cell or sensor used for the characterization

☒ Yes
☐ No

The light source in our lab is simulated AM 1.5 G illumination, enabled by a solar simulator (Newport IEC/JIS/ASTM) equipped with a 450 W xenon lamp and a Keithley 2400 source meter. It is calibrated by a commercial Si solar cell (1150 V, Newport Calibrated Reference Cell) before use. This information is provided in the 'Characterizations' section (probably incomplete) and will be further completed in subsequent revisions.

Explain why this information is not reported/not relevant.

Confirmation that the reference cell was calibrated and certified

☐ Yes
☒ No

Identify the independent certification laboratory.

The calibrator is a commercial Si solar cell (91150 V, Newport Calibrated Reference Cell). The information will be supplemented in further revisions.

Calculation of spectral mismatch between the reference cell and the devices under test

☐ Yes
☒ No

Provide a value of the spectral mismatch and/or a description of how it has been taken into account in the measurements.

The device performance is not only measured in our lab but also in an authoritative third-party institution, eliminating the potential impact of the possible spectral mismatch.

6. Mask/aperture

Size of the mask/aperture used during testing

☒ Yes
☐ No

The size determined by the mask is 0.0517 cm2 as presented in the certification report in Fig. S35.

Explain why this information is not reported/not relevant.

Variation of the measured short-circuit current density with the mask/aperture area

☐ Yes
☒ No

Report the difference in the short-circuit current density values measured with the mask and aperture area.

This information is irrelevant to the core of our report.

7. Performance certification

Identity of the independent certification laboratory that confirmed the photovoltaic performance

☒ Yes
☐ No

Fujian Metrology Institute, Fujian Province, China

Explain why this information is not reported/not relevant.

A copy of any certificate(s)

☒ Yes
☐ No

The certification report presented in Fig. S35.

Explain why this information is not reported/not relevant.

8. Statistics

Number of solar cells tested

☐ Yes
☒ No

Report how many solar cells have been tested, specifying the number of individual substrates.

Our work is not performance-focused, and this information is irrelevant to the core of our report.

Statistical analysis of the device performance

☐ Yes
☒ No

State where this information can be found in the text.

This information is irrelevant to the core of our report.

9. Long-term stability analysis

Type of analysis, bias conditions and environmental conditions

☒ Yes
☐ No

To support the scientific claim of this work, we carried out a fatigue J-V test of the device. The details can be found in the text relevant to Figure 4E. This information will be expanded in future revisions.

Explain why this information is not reported/not relevant.
